# Outer Membrane Vesicle Production Facilitates LPS Remodeling and Outer Membrane Maintenance in *Salmonella* during Environmental Transitions

**DOI:** 10.1128/mBio.01532-16

**Published:** 2016-10-18

**Authors:** Katherine E. Bonnington, Meta J. Kuehn

**Affiliations:** Department of Biochemistry, Duke University Medical Center, Durham, North Carolina, USA

## Abstract

The ability of Gram-negative bacteria to carefully modulate outer membrane (OM) composition is essential to their survival. However, the asymmetric and heterogeneous structure of the Gram-negative OM poses unique challenges to the cell’s successful adaption to rapid environmental transitions. Although mechanisms to recycle and degrade OM phospholipid material exist, there is no known mechanism by which to remove unfavorable lipopolysaccharide (LPS) glycoforms, except slow dilution through cell growth. As all Gram-negative bacteria constitutively shed OM vesicles (OMVs), we propose that cells may utilize OMV formation as a way to selectively remove environmentally disadvantageous LPS species. We examined the native kinetics of OM composition during physiologically relevant environmental changes in *Salmonella enterica*, a well-characterized model system for activation of PhoP/Q and PmrA/B two-component systems (TCSs). In response to acidic pH, toxic metals, antimicrobial peptides, and lack of divalent cations, these TCSs modify the LPS lipid A and core, lengthen the O antigen, and upregulate specific OM proteins. An environmental change to PhoP/Q- and PmrA/B-activating conditions simultaneously induced the addition of modified species of LPS to the OM, downregulation of previously dominant species of LPS, greater OMV production, and increased OMV diameter. Comparison of the relative abundance of lipid A species present in the OM and the newly budded OMVs following two sets of rapid environmental shifts revealed the retention of lipid A species with modified phosphate moieties in the OM concomitant with the selective loss of palmitoylated species via vesiculation following exposure to moderately acidic environmental conditions.

## IMPORTANCE

All Gram-negative bacteria alter the structural composition of LPS present in their OM in response to various environmental stimuli. We developed a system to track the native dynamics of lipid A change in *Salmonella enterica* serovar Typhimurium following an environmental shift to PhoP/Q- and PmrA/B-inducing conditions. We show that growth conditions influence OMV production, size, and lipid A content. We further demonstrate that the lipid A content of OMVs does not fit a stochastic model of content selection, revealing the significant retention of lipid A species containing covalent modifications that mask their 1- and 4′-phosphate moieties under host-like conditions. Furthermore, palmitoylation of the lipid A to form hepta-acylated species substantially increases the likelihood of its incorporation into OMVs. These results highlight a role for the OMV response in OM remodeling and maintenance processes in Gram-negative bacteria.

## INTRODUCTION

Gram-negative bacteria alter the composition of their outermost membrane in order to adapt to the disparate conditions encountered in various environmental niches (1, 2). Differences in the pH, osmolarity, and abundance of nutrients require constant fine-tuning of the bacterial envelope for maximal fitness. The abilities to respond to environmental changes and survive under stressful conditions are vital to bacterial proliferation and pathogenesis. In the hostile environment of a host organism, additional hazards, such as variable concentrations of reactive oxygen and nitrogen species, the presence of bile acids, and assault via antimicrobial peptides, necessitate appropriate and timely membrane changes by cells in order to retain proper barrier functionality (3).

Vast networks of two-component systems (TCSs) detect external conditions via inner membrane (IM) sensor kinases that can activate cognate transcription factors to promote and/or repress target gene expression (4). Two of the most extensively studied TCSs regulating outer membrane (OM) composition are PhoP/Q and PmrA/B. PhoP/Q responds to a low pH, a low magnesium concentration, and the presence of cationic antimicrobial peptides to activate genes encoding OM proteins, enzymes that covalently modify OM components, and the connector protein PmrD, which indirectly activates PmrA-regulated genes (3). In response to a mildly acidic pH, a high ferric iron level, and toxic aluminum concentrations, the PmrA/B TCS activates genes responsible for modulation of O-antigen length (*wzz_fepE_*, *wzz_ST_*), LPS core decorations (*pmrG*, *cptA*), and lipid A modifications (*pmrC*, *pmrR*, *pbgP*) (5). These modifications are known to be critical for *Salmonella* survival and replication, particularly during the periods of its pathogenic lifestyle when it resides within mammalian host cell lysosomes or has been engulfed by a phagocytic cell (6).

Certain characteristics central to the Gram-negative OM architecture and biogenesis complicate the process of rapid OM remodeling. For instance, the rate of lipopolysaccharide (LPS) diffusion in the OM is extremely low (7). Phospholipids may be degraded by phospholipases (e.g., PldA [8]) and recycled, but there is no known mechanism for LPS or outer membrane protein (OMP) degradation or recycling present in the OM (9). Though LPS molecules may be modified through the acylation activity of PagP (10), a very specific and unique OM-localized enzyme, other covalent modifications of LPS that are upregulated by environmental signals can only occur during the cytoplasmic process of LPS biosynthesis. Postsynthesis, these modified LPS species must then be incorporated into the OM through the stochastic process of OM biogenesis (11) to replace the “old” LPS in quantities sufficient to generate the beneficial phenotypic outcome (e.g., resistance to an otherwise deleterious environmental condition). Furthermore, once generated, sufficient levels of modified LPS species must be retained in order for the cell to derive maximal benefit from the energy-consuming biosynthetic process.

All of the Gram-negative bacteria studied to date produce OM vesicles (OMVs) (12). OMVs are the result of a constitutive process whereby sections of the OM bud outward from the peptidoglycan layer and pinch off to form spherical structures 10 to 200 nm in diameter (13). We considered that modulation of OMV production, as well as content, could affect the pace and maintenance of environmental change-induced OM remodeling. Additional selective enrichment of OMV LPS content for species detrimental to bacterial survival under current environmental conditions would further enhance the cell’s ability to change and/or maintain an environmentally ideal membrane composition.

In this study, we established a protocol in which bacteria change or maintain lipid A composition in a defined, reproducible native system. Utilizing this system, we examined OM and OMV compositions and measured OMV production levels. We compared experimentally determined relative ratios of LPS species to stochastic-model-based theoretical values in order to define the contribution of OMV production to the OM remodeling and maintenance processes of wild-type bacteria undergoing physiologically relevant changes in environmental conditions.

## RESULTS

### Development of a protocol for measuring environmentally controlled OM remodeling and maintenance.

In order to investigate the role of OMVs in the mediation and maintenance of OM remodeling, it was necessary to develop a protocol that would allow us to examine wild-type cells that are induced to accomplish a significant, measurable OM change in response to a physiologically relevant environmental shift but have a minimal opportunity to remodel their membrane via dilution upon cell growth and division. We further decided to focus on LPS since there are few known OM-localized enzymes (beyond the limited effect of PagP, PagL, and LpxR) that are known to act on this component after it has reached the OM.

**FIG 1:**
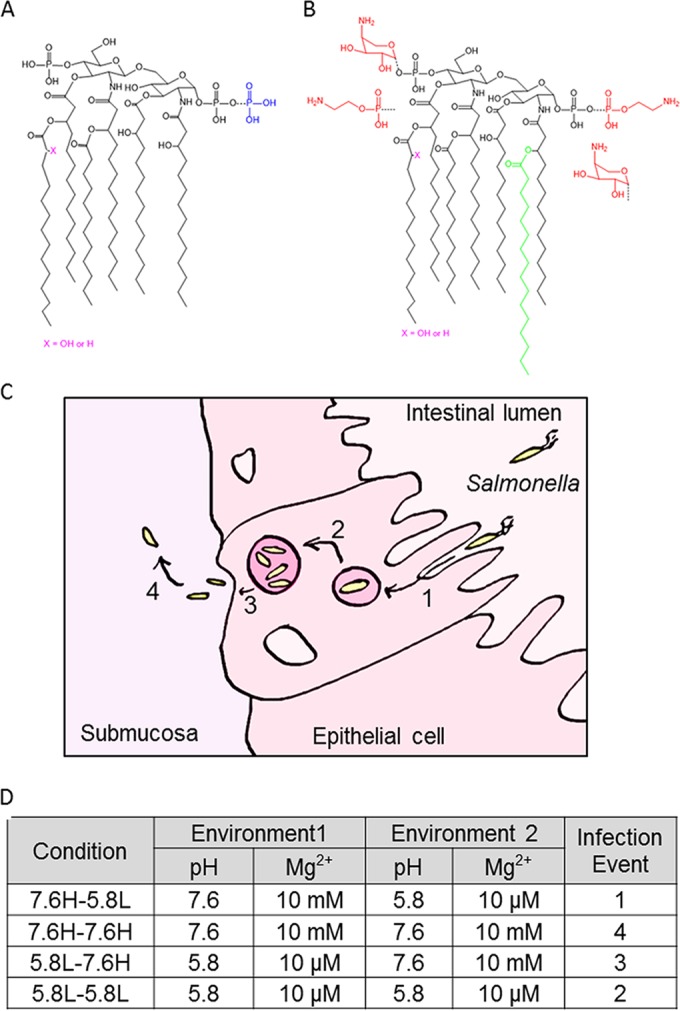
*Salmonella* lipid A structures present under different environmental conditions. The colored additions (blue, additional phosphate; red, pEtN and l-Ara4N; green, palmitoyl chain) to the base structure are representative of variations present at pH 7.6 10 mM Mg^2+^ (A) and pH 5.8 10 µM Mg^2+^ (B). (C) Diagram indicating the infection events mimicked by the environmental shift conditions. Salmonellae invade epithelial cells and become contained within an acidifying vacuole in step 1. Step 2 is *Salmonella* replication within this acidic SCV. The third step is *Salmonella* release into the submucosa, while the fourth step is survival and dissemination in this location. (D) The various environmental shift protocols are listed, with the high Mg^2+^ concentration corresponding to 10 mM MgSO_4_ and the low Mg^2+^ concentration corresponding to 10 µM MgSO_4_.

We utilized a well-established model system for PmrA/B- and PhoP/Q-repressing and -inducing conditions for *Salmonella enterica* serovar Typhimurium 14028S (14). In this model, two growth media differing in pH and divalent cation content simulate conditions present in the external environment and the lysosomal compartment. Substantial remodeling of the LPS, particularly its lipid A moiety, occurs upon the shift in environmental conditions (15). At pH 7.6 and 10 mM Mg^2+^ (7.6H), the lipid A subtypes present are hexa-acylated lipid A species: 1,4′-bisphosphate, 1-diphosphate, and 1-diphosphate, 4′-phosphate (Fig. 1A). The high concentration of divalent cations tightly bridges together the negatively charged phosphate moieties of lipid A molecules, creating a formidable permeability barrier (16). After a shift to pH 5.8 and 10 µM Mg^2+^ (5.8L), the cell’s membrane composition must rapidly adapt to the loss of magnesium ions, as the inability to bridge the acidic phosphate and pyrophosphate moieties of lipid A with divalent cations creates structural instability. Relief comes through the addition of positively charged 4-amino-4-deoxy-l-arabinose (l-Ara4N) and zwitterionic phosphoethanolamine (pEtN) groups to the phosphates of lipid A, which decrease the electrostatic repulsion between neighboring LPS molecules to stabilize OM bilayer structure (16). Similarly, palmitoylation of lipid A is believed to have a stabilizing effect on the bilayer structure by increasing hydrophobic interactions with neighboring LPS molecules (16). Accordingly, the number of lipid A subtypes present during the 7.6H-to-5.8L shift increased because of the gain of both covalent modifications and acyl chain derivatizations (Fig. 1B). The covalent modifications observed include one or two pEtN and/or l-Ara4N groups that are added to the 1 and/or 4′ phosphates (15). Although wild-type *S*. Typhimurium primarily modifies the 1 position of lipid A with pEtN when l-Ara4N synthesis is occurring, the enzymes that carry out these modifications possess the ability to modify both the 1 and 4′ phosphates with l-Ara4N and/or pEtN groups (15, 17). Derivatizations that co-occur with these modifications include the addition of a palmitoyl group to the acyl chain at position 2 (catalyzed by PagP) and/or the replacement of the secondary myristoyl chain at position 3′ with a 2-hydroxymyristoyl by LpxO (Fig. 1B). Modifications catalyzed by LpxR are not observed under these conditions because of the absence of calcium from the minimal medium; likewise, modifications catalyzed by PagL do not occur because of the inhibitory l-Ara4N modifications decorating the lipid A present under PhoP/Q-inducing conditions (18, 19).

For our quantitative *in vitro* experiments, we simulated the organism’s pathogenic lifestyle by shifting growth conditions to resemble the environmental challenges faced during epithelial cell invasion, replication, and dissemination (Fig. 1C). A defined minimal medium was used, as this has been demonstrated to result in the most reproducible rates of cell growth (2). *Salmonella* cultures were grown to mid-logarithmic phase (optical density at 600 nm [OD_600_], 0.6) in neutral, high-Mg^2+^ medium 7.6H (pH 7.6, 10 mM MgSO_4_), lightly centrifuged, and gently resuspended in fresh medium under either the same condition (7.6H) or shifted to fresh, mildly acidic, low-Mg^2+^ medium 5.8L (pH 5.8, 10 µM MgSO_4_) (Fig. 1D). Cells exposed to the reverse order of environmental conditions were grown to mid-logarithmic phase (OD_600_, 0.6) in 5.8L medium, lightly centrifuged, and gently resuspended in fresh medium with either the same composition (5.8L) or a different composition (7.6H) (Fig. 1D). The 7.6H-to-5.8L shift in conditions simulates changes encountered upon entry into the acidified *Salmonella*-containing vacuole (SCV), the 5.8L-to-5.8L mock shift in condition mimics survival and replication in an acidified SCV, the 5.8L-to-7.6H shift represents the changes upon release from an SCV, and the 7.6H-to-7.6H mock shift mimics the environment present during growth in the intestinal lumen, submucosa, or cytoplasm (Fig. 1C).

Cationic antimicrobial peptide resistance arises because of the OM remodeling that occurs in response to the stimuli present under the 5.8L condition (15). In order to determine what length of exposure to the 5.8L condition would result in a physiologically significant change in the amount of modified LPS in the OM, we assessed survival of a challenge with polymyxin B (PMB). We chose to examine low concentrations of PMB, between 2 and 20 µg/ml, known to increase the permeability of the OM and cause cell death without depolarizing the IM (20). Cultures were shifted from 7.6H to 5.8L conditions for 0, 45, or 90 min prior to treatment with 0, 4, or 8 µg/ml PMB (see Fig. S1A to C and Text S1 in the supplemental material). Notably, the cultures that experienced greater growth periods under the 5.8L condition displayed a higher percentage of survival at both 2 and 4 h after PMB addition. These data demonstrated that the OM changes occurring in the 90-min protocol reflect physiologically relevant and beneficial cellular events. We utilized this 90-min duration in all of our further analyses.

To perform direct comparisons of cells grown under the different shift protocols, the effect of the medium change on cell proliferation needed to be equivalent. Growth rate, cell morphology, and membrane integrity were characterized. Measurement of OD_600_ and CFU counts over time yielded very similar growth curves for all of the experimental conditions (see Fig. S2A and B in the supplemental material). The cultures doubled approximately two times over the course of the 90-min shift in conditions. Notably, the morphology of the 7.6H-grown cells did not significantly vary in length, diameter, or general appearance from cells grown or shifted to the 5.8L condition (see Fig. S3A to D in the supplemental material). Further, membrane integrity was not perturbed, as measured by Sytox Green uptake at multiple time points postshift (see Fig. S2C). Therefore, the data revealed no significant differences in cell viability or integrity dependent on environmental conditions that would confound the interpretation of results.

To further ensure comparability, the glycerophospholipid (GPL) and protein components in the OM of cells grown under each shift protocol were characterized in order to confirm that minimal changes occur during the initial 90 min of each environmental change. The *S*. Typhimurium OM GPL composition has been estimated to be 78% phosphatidylethanolamine (PE) and 22% phosphatidylglycerol (PG), acyl-PG, and cardiolipin (CL) (21). GPLs were isolated from the purified OM of cells that had undergone 90 min of 7.6H-to-7.6H, 7.6H-to-5.8L, 5.8L, or 5.8L-to-7.6H shift conditions before separation via two-dimensional (2D) thin-layer chromatography (TLC). A similar gross abundance of two primary GPLs, PE and PG, was observed in the OM under all of the conditions (see Fig. S3E to H in the supplemental material). In agreement with prior work demonstrating the regulatory role of PhoP/Q in increasing the levels of CL and palmitoylated acyl-PG within the OM, the OM isolated from the 7.6H-to-5.8L (shift to PhoP/Q-inducing) condition displayed a marked rise in acyl-PG and a slight increase in CL levels in comparison to those seen under the 7.6H-to-7.6H condition (see Fig. S3E and F in the supplemental material) (21). Exposure to mildly acidic low-Mg^2+^ medium (as in the 7.6H-to-5.8L, 5.8L-to-5.8L, and 5.8L-to-7.6H shifts) resulted in roughly comparable levels of both CL and acyl-PG between conditions. Although minor changes observed in OM-localized GPL head group composition must be considered in the comparison of cells undergoing the 7.6H-to-7.6H and 7.6H-to-5.8L shifts, GPL composition differences between the 5.8L-to-5.8L and 5.8L-to-7.6H conditions were largely indistinguishable.

Analogously, the protein compositions of OM samples purified from all of the conditions showed extensive similarity (see Fig. S3I in the supplemental material). However, the endpoint of the 7.6H-to-5.8L shift condition exhibited a greater abundance of an ~37-kDa band (likely because of acidic-pH-induced OmpC expression) and the presence of ~23- and ~17-kDa bands absent from the 7.6H-to-7.6H condition OM. The differences between the 5.8L-to-5.8L and 5.8L-to-7.6H conditions were minor; the intensity of the ~37-kDa band (OmpF/C) decreased and the ~17-kDa band disappeared because of the shift in environment. Accordingly, alterations in OMP composition minimally impact the comparison of the two sets of environmental shift conditions.

### Monitoring the lipid A composition of the OM.

**FIG 2:**
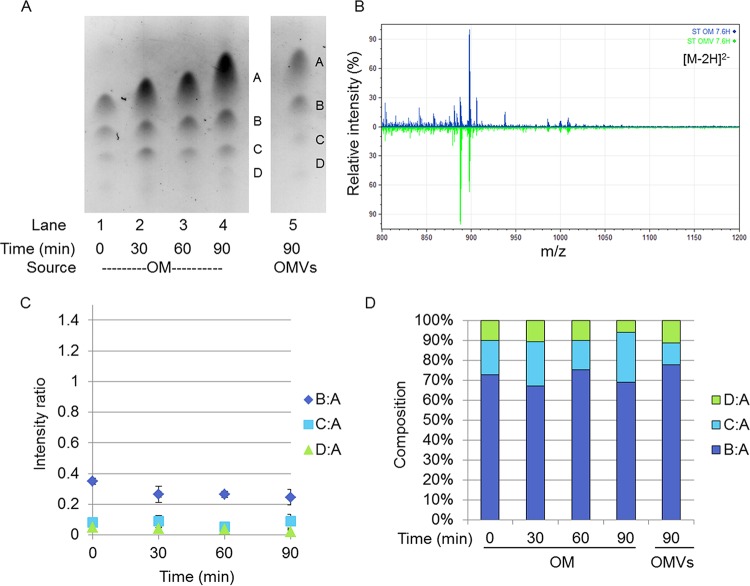
Comparison of OM and OMV lipid A compositions under the 7.6H-to-7.6H condition. (A) Representative TLC separation of lipid A from WC time points (OM) and OMV from the 7.6H-to-7.6H condition. (B) Negative-ion ESI-MS showing the doubly charged lipid A species from 7.6H-to-7.6H OM (top) and OMV (bottom) 90-min time points. (C) Values of the ratios derived from densitometric quantification of the bands present in TLC separations. All ratios are in reference to band A. (D) Representation of densitometric ratios as percentages of the total composition. The ratios presented in panel C are percentages of the total sum of all of the ratios present per time point. The standard errors of the composition measurements at the different time points are shown in Fig. S6A in the supplemental material.

**FIG 3:**
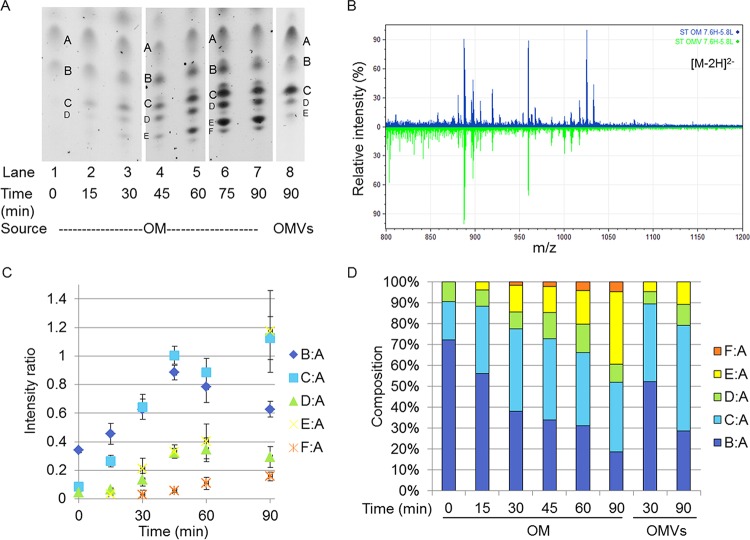
Comparison of OM and OMV lipid A compositions under the 7.6H-to-5.8L condition. (A) Representative TLC separation of lipid A from WC time points (OM) and OMVs from the 7.6H-to-5.8L condition. Some lanes have been adjusted in overall contrast for this presentation so the images can be more easily compared, but unaltered images were used during quantification. (B) Negative-ion ESI-MS showing the doubly charged lipid A species from the 7.6H-to-5.8L OM (upper) and OMVs (lower) at the 90-min time point. (C) Values of the ratios derived from densitometric quantification of the bands present in TLC separations. All ratios are in reference to band A. (D) Representation of the densitometric ratios as percentages of the total composition. The ratios presented in panel C are percentages of the total sum of all of the ratios present per time point. The standard errors of the composition measurements at the different time points are shown in Fig. S6B in the supplemental material.

**FIG 4:**
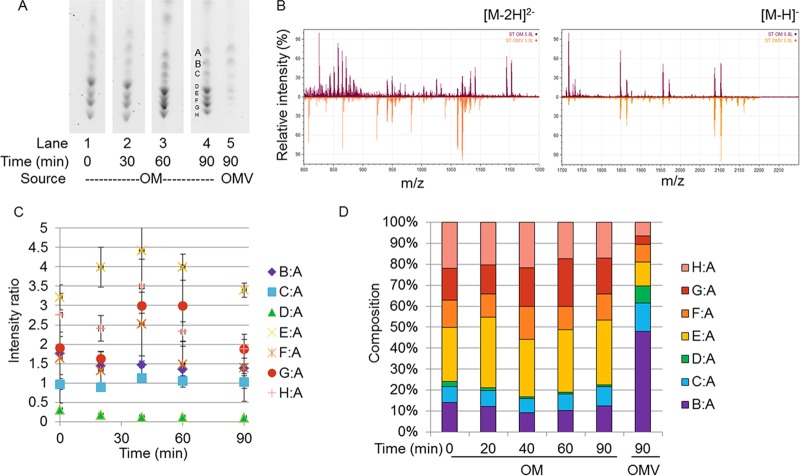
Comparison of OM and OMV lipid A compositions under the 5.8L-to-5.8L condition. (A) Representative TLC separation of lipid A from WC time points (OM) and OMVs from the 5.8L-to-5.8L condition. (B) Negative-ion ESI-MS showing the doubly (*m*/*z* 800 to 1,200) and singly (*m*/*z* 1,700 to 2,200) charged lipid A species from the 5.8L-to-5.8L OM (upper) and OMVs (lower) at the 90-min time point. (C) Values of the ratios derived from densitometric quantification of the bands present in TLC separations. All ratios are in reference to band A. (D) Representation of densitometric ratios as percentages of the total composition. The ratios presented in panel C are percentages of the total sum of all of the ratios present per time point. The standard errors of the composition measurements at the different time points are shown in Fig. S6C in the supplemental material.

**FIG 5:**
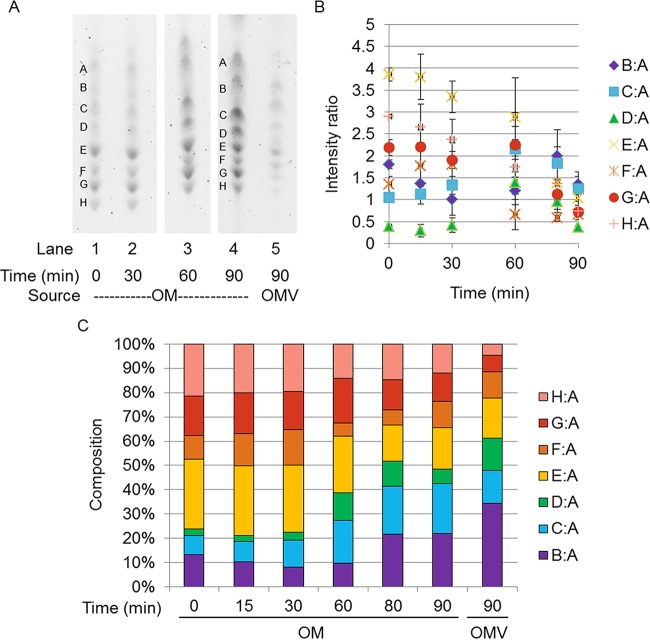
Comparison of OM and OMV lipid A compositions under the 5.8L-to-7.6H condition. (A) Representative TLC separation of lipid A from WC time points (OM) and OMVs from the 5.8L-to-7.6H condition. (B) Values of the ratios derived from densitometric quantification of the bands present in TLC separations. All ratios are in reference to band A. (C) Representation of the densitometric ratios as percentages of the total composition. The ratios presented in panel B are percentages of the total sum of all of the ratios present per time point. The standard errors of the composition measurements at the different time points are shown in Fig. S6D in the supplemental material.

OM lipid A structures of growing cultures were assessed at 15- to 30-min intervals over the 90 min following the medium change (Fig. 2A, 3A, 4A, and 5A; see Fig. S4A in the supplemental material). Lipid A was purified from whole-cell (WC) samples via the standard chloroform-methanol extraction and hydrolysis method prior to separation by TLC and visualization by charring. In this TLC system, analogous to those used previously (14), three bands represent the primary lipid A species present in the OM under the 7.6H condition (Fig. 2A and 3A; see Fig. S4A in the supplemental material). In contrast, more numerous lipid A species were present in cultures shifted from or to 5.8L medium (Fig. 3A, 4A, and 5A). Notably, the number of lipid A bands increased fairly rapidly (to approximately seven) by 60 min after the shift from 7.6H to 5.8L medium (Fig. 3A; see Fig. S4A in the supplemental material), whereas upon a reverse 5.8L-to-7.6H shift in conditions, a modest and slow change in lipid A composition occurred, with only a gradual intensification of upper bands apparent over the 90-min time period (Fig. 5A).

In previous work, the various lipid A species were assigned group numbers (14) to indicate both the structures and the separation patterns these species take on after analysis by TLC (see Fig. S4A and C in the supplemental material). These group numbers (1 to 4) are indicated in the results where appropriate.

Mass spectrometry (MS) was used to identify the lipid A species present after the 90-min shift in each condition. Three hexa-acylated lipid A species, 1,4′-bisphosphate (1,798/1,814 Da; Group 2), 1-diphosphate (1,718/1,734 Da; Group 1), and 1-diphosphate, 4′-phosphate (1,878 Da; Group 3), were confirmed to be present during the 7.6H-to-7.6H mock shift (see Fig. S4C in the supplemental material; Table 1). At 90 min after a 7.6H-to-5.8L shift, in addition to lipid A species seen in the 7.6H-to-7.6H mock shift (with the exception of the 1-diphosphate, 4′-phosphate species), the hexa-acylated lipid A species detected were covalently modified with pEtN and/or l-Ara4N or a hepta-acylated derivative of the base lipid A 1,4′-bisphosphate with a palmitoyl group attached to the position 2 acyl chain (2,037 Da; Group 2) (see Fig. S4C in the supplemental material; Table 1). The covalent modifications observed were as follows: a single pEtN attached to either the 1- or 4′-phosphate (1,841/1,857 Da, 1,921/1,937 Da; Group 3), a single l-Ara4N attached to the 1- or 4′-phosphate (1,930/1,946 Da; Group 3), and one pEtN and one l-Ara4N attached at the 1- and 4′-phosphates (2,053/2,069 Da; Group 4) (see Fig. S4C in the supplemental material; Table 1). The major species were all hexa-acylated: the base lipid A structure, lipid A modified with 1- or 4′-P-pEtN, and a 1-P-pEtN, 4′-P-l-Ara4N lipid A species (Table 1).

**TABLE 1:** Cellular lipid A composition after 90 min of environmental shift

Growth condition	*M*_r_^a^	[M-H]^−^_p_^b^ (*m*/*z*)	No. of groups at 1 and/or 4′ position				No. of acyl chain additions	
			P^c^	di-P^d^	P-pEtN^e^	P-l-Ara4N^f^	C_16:0_^g^	OH^h^
7.6H to 7.6H	1,718	1,715	1					
	1,798	1,775	2					
	1,798	1,796	2					
	1,813	1,811	2					1
	1,878	1,876	1	1				
7.6H to 5.8L	1,798	1,775	2					
	1,798	1,796	2					
	1,813	1,811	2					1
	1,841	1,838			1			
	1,856	1,854			1			1
	1,921	1,918	1		1			
	1,929	1,926	1			1		
	2,036	2,033	2				1	
	2,053	2,049			1	1		
	2,068	2,065			1	1		1
5.8L to 5.8L	1,718	1,716	1					
	1,734	1,732	1					1
	*1*,*798*	1,751	2					
	*1*,*798*	1,767	2					
	1,798	1,796	2					
	1,850	1,847				1		
	1,865	1,863				1		1
	1,878	1,882	1	1				
	1,894	1,898	1	1				1
	1,957	1,954					1	
	2,088	2,086				1	1	
	2,103	2,101				1	1	1
	2,117	2,120	1	1			1	
	2,133	2,136	1	1			1	1
	2,160	2,161	1		1		1	
	2,175	2,180	1		1		1	1
	2,283	2,287			2		1	
	2,298	2,303			2		1	1

a The *M*_r_ is the calculated molecular weight of the proposed structure; italicized values indicate less certainty in assignments.

b ^−^_p_ is the predicted value for singly charged MS ions.

c P, unsubstituted monophosphate.

d di-P, unsubstituted diphosphate; at the 1 position.

e P-pEtN, diphosphoethanolamine; at either the 1 or the 4′ position but likely at the 1 position when l-Ara4N is present.

f P-l-Ara4N, phospho-l-Ara4N.

g C_16:0_, palmitate addition mediated by *pagP.*

h OH, in 2-hydroxymyristate mediated by *lpxO.*

Unlike the culture analyzed 90 min after a shift to 5.8L medium (7.6H to 5.8L), in cultures starting from the 5.8L condition (either undergoing a mock shift [5.8L to 5.8L] or a shift [5.8L to 7.6H] in conditions), the cells have had additional time to incorporate further-modified species into the OM. Consequently, these featured many singly and doubly pEtN-containing species (2,161/2,180 and 2,287/2,303 Da), a few l-Ara4N-containing species (1,850/1,865, 1,930, and 2,103 Da), and a plethora of palmitoylated species (1,957, 2,103, 2,117/2,133, 2,161/2,180, and 2,287/2,303 Da) (see Fig. S4C in the supplemental material; Table 1). Interestingly, 1-diphosphate, 4′-phosphate species were in moderate abundance (1,978/1,894 and 2,117/2,133 Da, Group 3) (see Fig. S4C in the supplemental material; Table 1). The major species in the 5.8L cultures were all hexa-acylated lipid A: 1,4′-bisphosphate; 1-diphosphate, 4′-phosphate; and a singly P-l-Ara4N modified structure.

### Quantification of OM- and OMV-localized lipid A species.

In order to investigate the possibility that OM vesiculation could enhance OM remodeling, we needed to analyze the lipid A composition of OMVs produced explicitly over the duration of the environmental shifts. To accomplish this, large volumes of bacterial culture were necessary to obtain sufficient amounts of OMV material to yield the quantities of purified lipid A needed to perform compositional analysis of lipid A subspecies. Because of this constraint, standard methods of ^32^P labeling and subsequent quantitative TLC analysis could not be feasibly employed. Instead, we performed densitometric analysis of the lipid A species present in the TLC bands observed by the charring method.

For ease of quantitation, bands were labeled A to F, with partially overlapping bands grouped into a single letter category (see Fig. S4A in the supplemental material). We were aware that the intensity of each band does not directly correspond to the amount of material per sample, as different species may char at slightly different rates and inconsistent yields of lipid A may occur per set amount of starting material in purification. Therefore, we controlled internally for these factors by calculating the ratio of an individual band to band A, a band present in all of the samples analyzed. The reproducibility of this method was confirmed for dilutions of both simple and complex samples with biological and technical replicates (see Fig. S5A and B in the supplemental material). Additionally, since all of the TLC plates analyzed contained an OM sample prepared from the initial (*t* = 0 min) culture, the consistency of the charring method between different plates could be further verified.

Correlations between the separation pattern and intensity of bands present in our TLC system and the relative abundance of lipid A species present in a series of samples by MS allowed us to assign the bands to Groups 1 to 4 developed by H. S. Gibbons et al. (14) (see Fig. S4A and C and Table S1 in the supplemental material). Accordingly, we strongly suspect that the high intensity of band A on TLC plates (Fig. 2A) corresponds to the abundance of the unmodified 1,4′-bisphosphate lipid A species (Group 2) observed by electrospray ionization (ESI)-MS (Fig. 2B; Table 1), making it an appropriate denominator for comparisons. Likewise, the comparison of the intensities of bands E and F in the 90-min 7.6H-to-5.8L OMV and OM samples (Fig. 3A), coupled with the respective abundance/absence of the doubly modified lipid A species (Fig. 3B; see Table S1 in the supplemental material), led us to assign these bands to Group 4. Confirmation of these assignments was obtained by staining for nitrogen-containing groups with ninhydrin (see Fig. S4B in the supplemental material). As expected, we observed the presence of nitrogenous modifications in the lower bands at the 90-min time point under the 7.6H-to-5.8L and 5.8L-to-5.8L conditions, but not the 7.6H-to-7.6H condition. As bands C and D appear under both the 7.6H-to-7.6H and 7.6H-to-5.8L conditions after charring but only under the 7.6H-to-5.8L condition after ninhydrin staining, these were placed into diverse Group 3, containing lipid A species modified with diphosphate, a single P-pEtN, or P-l-Ara4N (see Fig. S4C in the supplemental material). Group 1 species, which lose phosphate moieties during the purification process, were confirmed through the lesser intensity of these higher bands by malachite green staining than by charring (data not shown).

### OMV-localized lipid A species are somewhat depleted of modified species during the shift from 7.6H to 5.8L conditions.

As expected, over the 90-min duration of the 7.6H-to-7.6H mock shift, the lipid A composition of the OM remained static (Fig. 2A and C). The B:A ratio hovered around 71.00% ± 9.35%, the C:A ratio was 19.79% ± 7.15%, and the D:A ratio was approximately 9.22% ± 2.88% (Fig. 2D; see Fig. S6A in the supplemental material). The composition of the OMVs produced over 90 min of the 7.6H-to-7.6H mock shift in conditions closely mirrored the state of the OM over this time: 77.64% ± 33.02% B:A, 11.03% ± 0.29% C:A, and 11.33% ± 0.21% D:A (Fig. 2D). By ESI-MS, the 1,4′-bisphosphate (1,798 Da, *m*/*z* 898 [M-2H]^2−^) and 1-diphosphate hexa-acylated lipid A structures (1,878 Da, *m*/*z* 939 [M-2H]^2−^) displayed identical relative intensities in the 7.6H-to-7.6H OM and OMVs (Fig. 2B).

In contrast, over the 90 min of the 7.6H-to-5.8L shift in conditions, the lipid A composition of the OM changed markedly as additional Group 3 and 4 species were synthesized (Fig. 3A and B; Table 1). Accordingly, B:A decreased to 18.59% ± 1.66% of the total and C:A peaked at 30 min postshift before leveling out at 33.34% ± 4.48% after 90 min (Fig. 3D; see Fig. S6B in the supplemental material). The D:A ratio, representing Group 3 lipid A species, increased before plateauing around 45 min, but because of fluctuations in the abundance of other species, this particular portion made up a relatively stable percentage of the total species, staying in the range of 8.09% ± 2.74 to 13.61% ± 3.30% over the 90-min time period (Fig. 3C and D). The ratios of bands making up Group 4, E:A and F:A, both increased over the course of the 7.6H-to-5.8L shift. Band E began to appear at only 15 min postshift, and by 90 min, E:A made up 34.78% ± 8.52% of the total. B and F initially appeared later, at 30 min post shift, and F:A only increased to 4.66% ± 0.87% of the total species by 90 min (Fig. 3D; see Fig. S6B in the supplemental material).

Notably, the composition of the OMVs collected during the first 30 min of the shift from 7.6H to 5.8L conditions resembled the state of the OM at 15 min. The 30-min OMVs were composed of 52.40% ± 4.37% B:A, 36.96% ± 15.45% C:A, 5.87% ± 2.79% D:A, and 4.78% ± 0.61% E:A (Fig. 3D). The lipid A content of the OMVs produced over 90 min of the 7.6H-to-5.8L shift condition best resembled the OM at a time between 30 and 45 min. The 90-min OMVs were composed of 28.2% ± 4.6% B:A, 50.40% ± 9.67% C:A, 9.79% ± 0.40% D:A, and 10.98% ± 1.69% E:A (Fig. 3D). The percentage of E:A nearly tripled in the OM from the 30-min time point to the 90-min time point. For the OMV composition, the abundance of E:A only doubled when the OMVs collected over 90 min were compared to those collected over 30 min. Notably, band F was undetectable after 30 and 90 min of OMV production under the 7.6H-to-5.8L shift condition.

Comparison of the lipid A composition of the final OM time point with that of the OMVs produced over the 90 min of the 7.6H-to-5.8L shift revealed major differences in the relative intensities of singly and doubly modified lipid A species (Fig. 3B; see Table S1 in the supplemental material). Lipid A structures modified with a single P-pEtN (1,841/1,856, 1,921 Da; Group 3) were present in the OM at approximately twice the intensity found in the OMVs. Similarly, lipid A species modified with a single P-l-Ara4N (1,929 Da; Group 3) were observed in small quantities in the OM but were not detected in the OMVs. Most strikingly, lipid A species decorated with both P-l-Ara4N and P-pEtN (2,053/2,068 Da; Group 4) were seen in large quantities in the 90-min 7.6H-to-5.8L OM but were not detected in the OMVs produced over the duration of the environmental shift. These results suggest that OMVs were depleted of LPS species induced upon the shift from 7.6H to 5.8L conditions, which could indicate a positive role for OMVs in enabling OM remodeling toward an environmentally adapted LPS composition.

In regard to acylation changes, the only lipid A-targeted PagP activity detected within the 90 min of the 7.6H-to-5.8L environmental shift was the palmitoylation of 1,4′-bisphosphophate lipid A (Fig. 3B; see Table S1 in the supplemental material). Though the hepta-acylated 1,4′-bisphosphate lipid A species (2,036 Da; Group 2) was not observed in the OM of the 7.6H-to-5.8L shift until after the 60-min time point, this palmitoylated lipid A displayed similar intensity in both the 90-min OM and OMV samples (see Table S1 in the supplemental material). These data confirm findings by another group (22) in which a similar abundance of hepta-acylated 1,4′-bisphosphorylatied lipid A was observed in both the OM and OMVs of *S*. Typhimurium grown in Luria broth (LB), a medium depleted of divalent cations.

### OMV-localized lipid A species are substantially depleted of modified species during the shift from 5.8L to 7.6H conditions.

Over the 90-min time period of the 5.8L-to-5.8L mock shift, the lipid A composition of the OM remained relatively static, as expected (Fig. 4A and C; see Fig. S6C in the supplemental material). The B:A ratio hovered around 11.57% ± 1.80%, the C:A ratio was 7.88% ± 0.74%, the D:A ratio was 1.25% ± 0.16%, the E:A ratio was 29.49% ± 3.44%, the F:A ratio was 12.71% ± 6.17%, the G:A ratio was 17.40% ± 4.11%, and the H:A ratio was 19.70% ± 2.74% (Fig. 4D; see Fig. S6C in the supplemental material). In the cultures undergoing a shift from 5.8L to 7.6H conditions, we observed a decreased abundance of modified lipid A species over time, although the onset of the change was delayed compared to that in the 7.6H-to-5.8L protocol (Fig. 5A and B). Significantly, the late Group 4 G:A and H:A ratios fell to 11.75% ± 5.64% and 11.90% ± 6.03% of the total species, with the majority of the decrease occurring after 60 min of growth in the new 7.6H environment (Fig. 5C; see Fig. S6D in the supplemental material). The early Group 4 ratios experience a similar trend; E:A levels decreased to 17.00% ± 0.98% and F:A levels hovered around 11.18% over the entirety of the 5.8L-to-7.6H shift time period. Concurrently, the Group 1-populated B:A ratio swelled from approximately 10.41% to 22.00% ± 3.48%, with most of this increase occurring in the last 30 min of the 5.8L-to-7.6H shift. The Group 3 C:A and D:A levels both increase gradually by 2- to 3-fold over the 90 min to become 20.44% ± 12.55% and 6.11% ± 4.18% of the total species, respectively (Fig. 5C; see Fig. S6D in the supplemental material).

Surprisingly, the composition of the OMVs collected from both the 5.8L-to-7.6H shift and 5.8L-to-5.8L mock shifted cells did not closely resemble the state of the OM of the cells from either condition. The 90-min 5.8L-to-5.8L OMVs were composed of 48.00% ± 1.44% B:A, 13.46% ± 0.30% C:A, 8.13% ± 7.05% D:A, 11.46% ± 4.64% E:A, 8.36% ± 4.57% F:A, 4.09% ± 0.50% G:A, and 6.50% ± 0.92% H:A, and the 5.8L-to-7.6H OMVs were composed of 34.28% ± 7.11% B:A, 13.70% ± 2.11% C:A, 13.21% ± 10.3% D:A, 16.67% ± 6.82% E:A, 10.61% ± 6.73% F:A, 6.82% ± 2.15% G:A, and 4.72% ± 3.89% H:A (Fig. 4D and 5C). The Group 3 lipid A species present in bands G and H were significantly less abundant in the 5.8L-to-5.8L and 5.8L-to-7.6H OMVs than in the OM at the respective time points. Depletion of these species in the OMVs is at the expense of the less modified Group 1 and 2 species present in band B, as these lipid A species make up a significantly higher percentage of the OMV composition than in the OM at all time points (0 to 90 min) following the 5.8L-to-5.8L and 5.8L-to-7.6H shifts. These results indicate that OMVs produced under 5.8L conditions were substantially depleted of the specifically modified LPS species, even after a shift back to 7.6H.

To determine the exact species experiencing enriched incorporation into OMVs under this environmental condition, ESI-MS analysis revealed the relative intensities of lipid A species isolated from the OM and OMVs (see Table S1 in the supplemental material). Notably, 1-diphosphate, 4′-phosphate hepta-acylated lipid A derivatives (2,117/2,133 Da), present in small amounts in the 5.8L-to-5.8L OM, was the most abundant species of 5.8L-to-5.8L OMVs. Conversely, 1,4′-bisphosphate (1,734 to 1,798 Da) and 1,4′-bisphosphoethanolamine hexa-acylated lipid A species (2,283/2,298 Da), the predominant species of the 5.8L-to-5.8L OM, experienced undetectable incorporation into OMVs. Hepta- and hexa-acylated P-l-Ara4N-modified lipid A species (1,850/1,865, 2,088/2,103 Da) displayed comparable levels of intensity in OMVs and the OM (see Table S1 in the supplemental material).

### OMV production increases during the shift to 5.8L medium.

**FIG 6:**
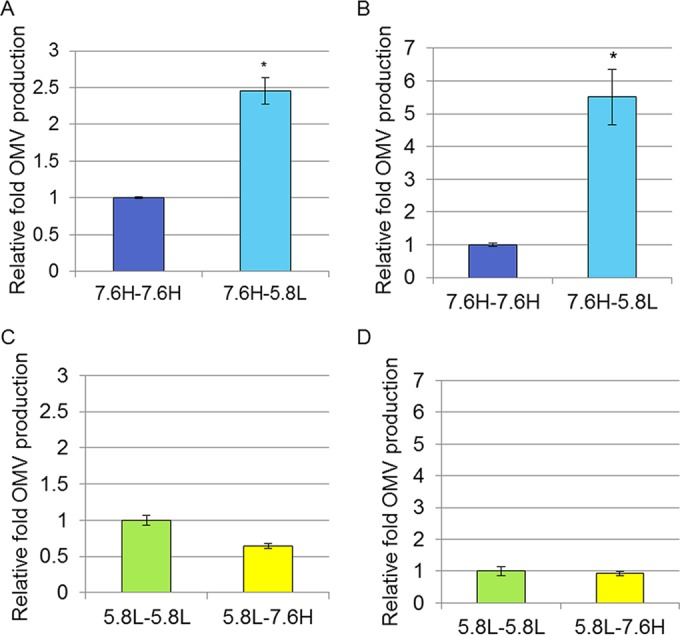
OMV production under environmental shift conditions. The relative OMV production, normalized to the CFU count, during the 90-min period under the 7.6H-to-7.6H and 7.6H-to-5.8L (A and B) and 5.8L-to-5.8L and 5.8L-to-7.6H (C and D) conditions, as calculated for protein content by OMP densitometry (A and C) and lipid content by FM4-64 fluorescence (B and D). For all conditions, *n* >3; *, statistically significant differences (*P* < 0.05) between production values for the pairs of conditions indicated.

The relative concentrations of OMVs present in the cell-free supernatants of cultures from each condition were determined by measuring protein content via OMP densitometry and lipid content via fluorescence from the lipophilic dye FM4-64 as described in a prior report (23). Both methods found OMV production to be significantly higher, 2.5-fold by protein measurement and 6-fold by lipid measurement, in cultures undergoing the 7.6H-to-5.8L shift than in those undergoing the 7.6H-to-7.6H mock shift in conditions (Fig. 6A and B). The protein compositions of the OMVs collected after both shifts in conditions were highly similar (see Fig. S7B in the supplemental material).

The disparity between FM4-64 and OMP densitometry measurements in the fold increase of 7.6H-to-5.8L shift over 7.6H-to-7.6H mock shift OMV production lead us to believe that the OMVs produced under the shift condition may have more lipid material per protein content than those produced in the mock shift. In order to test this prediction, the density profiles of the two OMV populations were determined by equilibrium gradient centrifugation (see Fig. S7A in the supplemental material). The density of the 7.6H-to-7.6H OMVs resembles a sharp, symmetric distribution, with the bulk of the OMV material (>60%) residing in fractions 6 and 7. The distribution of the 7.6H-to-5.8L OMVs is both more broad and shifted toward the lighter side of the gradient, with a peak around fraction 6 and >90% of the material in fractions 4 to 7.

### OMV production initially remains high during the shift from 5.8L to 7.6H but later reverts to basal levels.

Unlike the upregulation of OMV production in the shift from 7.6H to 5.8L compared to the 7.6H-to-7.6H mock shift, OMV production by 5.8L cultures shifted to 7.6H or mock shifted to 5.8L conditions did not differ greatly over the 90-min time period (Fig. 6C and D). OMV production during the shift from 5.8L to 7.6H was 0.64-fold that of the 5.8L-to-5.8L mock shift in condition, as measured via OMP densitometry, but was not significantly different from the 5.8L-to-5.8L mock shift in condition as measured by FM4-64 fluorescence.

Over this short time period, the protein composition of the secreted OMVs under both conditions did not visibly differ (see Fig. S7B in the supplemental material). However, we did note that upon overnight growth post shift for both 5.8L-to-7.6H and 7.6H-to-5.8L cultures, the abundance of acidic-pH-induced OMPs present in the OMVs changed, and OMV production of 5.8L-to-7.6H cultures returned to the lower levels observed under the 7.6H-to-7.6H mock shift and 7.6H overnight growth conditions (data not shown).

The separate effects of changes in Mg^2+^ concentration and pH on OMV protein and lipid were also studied. OMV production upon overnight growth at pH 7.6, regardless of whether the concentration of Mg^2+^ was high or low, was significantly lower than OMV production in pH 5.8 medium, as determined by OMPs (see Fig. S7D in the supplemental material). However, the production of OMVs as determined by FM4-64 for 5.8L-grown overnight cultures was as low as that for 7.6H/L-grown cultures (see Fig. S7E in the supplemental material). These results suggest that induction of increased levels of OMVs is a long-term effect initiated by the mildly acidic pH and that the Mg^2+^ concentration may influence the ratio of lipid to protein material packaged into the OMVs and/or the accessibility of the OMV surface to FM4-64 intercalation.

### Growth under acidic conditions results in increased OMV size.

**FIG 7:**
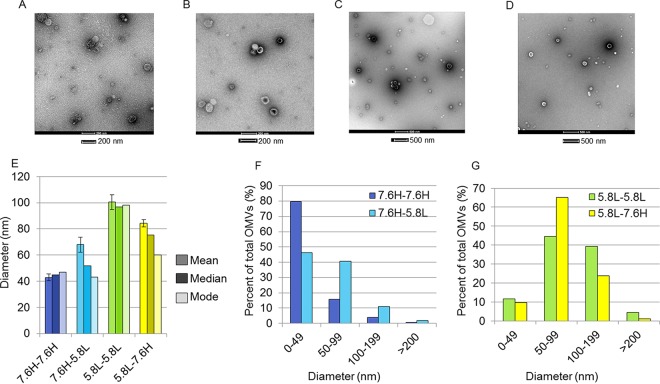
OMV morphology under environmental shift conditions. Shown are representative TEM micrographs of negatively stained OMVs collected from 90 min under the 7.6H-to-7.6H (A), 7.6H-to-5.8L (B), 5.8L-to-5.8L (C), and 5.8L-to-7.6H (D) conditions. Size bars, 200 nm (A and B) and 500 nm (C and D). (E) Mean, median, and mode of diameters of OMVs from each environmental shift condition (over three different micrographs, *n* ≥55). (F and G) Distribution of the measured OMV diameters under the 7.6H-to-7.6H and 7.6H-to-5.8L conditions (F) and the 5.8L-to-5.8L and 5.8L-to-7.6H conditions (G).

In order to further assess the disparity between protein and lipid measurements of OMVs produced upon a shift, we examined negatively stained transmission electron microscopy (TEM) images of OMVs. OMVs collected from 7.6H-to-7.6H mock shifted and 7.6H-to-5.8L shifted cultures (Fig. 7A and B) revealed gross morphological similarities with slight differences in size. The mean diameter of OMVs produced during the 7.6H-to-5.8L shift protocol was at least 20 nm larger: 68 ± 5.67 nm (*n* = 55) for the 7.6H-to-5.8L shift versus 43 ± 2.63 nm (*n* = 133) for the 7.6H-to-7.6H mock shift (Fig. 7E). Indeed, the size distribution of the OMVs produced during the 90-min 7.6H-to-7.6H mock shift skewed toward the smaller sizes (Fig. 7F).

To determine whether the size distributions of the OMVs produced in 5.8L-to-7.6H shifted or 5.8L-to-5.8L mock-shifted cultures differed in response to environmental change or simply possess different properties under various environmental conditions, we assessed the morphology and size of the 5.8L-to-5.8L mock shifted and 5.8L-to-7.6H shifted OMVs via TEM (Fig. 7C and D). The morphology of these OMVs was similar to that of the 7.6H-to-7.6H and 7.6H-to-5.8L OMVs; however, the size distribution under both conditions was shifted to the right, with the average diameters of the 5.8L-to-5.8L mock shifted and 5.8L-to-7.6H shifted OMVs measured as 100.43 ± 5.7 nm (*n* = 112) and 84.16 ± 2.7 nm (*n* = 184), respectively (Fig. 7E and G). Therefore, greater time spent in the mildly acidic medium correlates with a larger diameter of the OMVs produced under each growth condition (in the order of 5.8L to 5.8L [5.5 h], 5.8L to 7.6H [4 h], 7.6H to 5.8L [1.5 h], and 7.6H to 7.6H [0 h]). Additionally, we observed a greater variation in the mean, median, and mode of the measured diameter of OMVs produced during an environmental change (the 7.6H-to-5.8L or 5.8L-to-7.6H shift condition) than under the corresponding mock shift conditions, where the three parameters did not significantly differ (Fig. 7E).

### The observed OMV composition does not support a stochastic model of OMV production under all environmental conditions.

If packaging of lipid A species into OMVs were a completely stochastic process, then the composition of the OMVs produced over the 90 min of the environmental shift protocols would be expected to exactly mirror the composition of the OM material over time. For this model we must assume that vesiculation occurs at a constant rate fixed to cell growth, that LPS can mix ideally, and that neither protein contents nor the physical properties of LPS molecules affect OMV formation.

**FIG 8:**
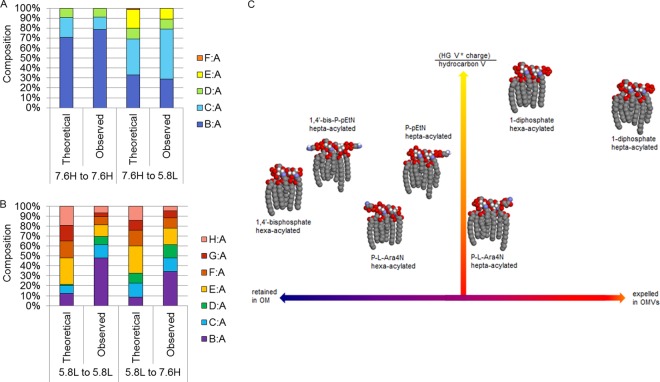
Comparison of experimentally determined OMV composition by using theoretical stochastic models. The percent composition of each lipid A species ratio is shown. The observed experimental data are shown next to theoretical values based upon the composition of the OM over time under each condition. (A) Experimental 7.6H-to-7.6H OMV compositions display the least difference from the predicted composition, whereas the 7.6H-to-5.8L values deviate somewhat from the respective predicted compositions. (B) Experimental 5.8L-to-5.8L and 5.8L-to-7.6H OMV compositions deviate substantially from the predicted respective compositions. (C) The seven main lipid A structures present during the 5.8L-to-5.8L shift are shown here as three-dimensional space-filling models (in typical Corey-Pauling-Koltun coloring, with the majority of the H atoms not shown for simplicity). The placement of each molecule on the *x* axis represents its propensity to be retained in the OM (left, blue), expelled in OMVs (right, red), or neither (center, purple), as estimated from the ESI-MS data presented in Table S1 in the supplemental material. A calculation combining estimations for the van der Waals volume of each molecule’s head group (HG V) and acyl chains (hydrocarbon V), along with the net charge (absolute value used in calculation) of each molecule was used to place the molecules along the *y* axis. The values for the HGV*|charge|/HCV ratio are shown from low (red) to high (yellow).

To obtain values for the total abundance of each band of lipid A subtypes created over time, we fitted each relevant lipid A ratio (B:A to H:A) to curves that modeled the progression of membrane lipid A composition. Integration of the curves representing the continuous values of each lipid A component allowed for the formulation of the theoretical composition of OMVs displayed in Fig. 8. We compared the values we derived from this model to our experimental results in order to test if this stochastic model could account for the lipid A composition we observed in the OMVs produced under each condition.

As the predicted lipid A composition of the 7.6H-to-7.6H mock shift OMVs closely resembled the OM compositions displayed in Fig. 2C, the experimentally determined lipid A composition of these mock shift OMVs matched the stochastic model closely (Fig. 8A). However, a slightly higher percentage of the B:A ratio was observed in the experimentally determined composition (see Fig. S8A in the supplemental material). The observed and theoretical lipid A compositions of the 7.6H-to-5.8L OMVs exhibited substantial differences only in the levels of Group 4 species, whereas the other species were mostly in agreement (Fig. 8A). The abundance of both the E:A and F:A ratios was less than that predicted by the progression of the OM change (see Fig. S8A in the supplemental material).

Intriguingly, for the 5.8L-to-5.8L condition, the theoretical model of OMV lipid A composition was vastly different from the experimentally observed composition (Fig. 8B). The B:A abundance observed in the 5.8L-to-5.8L mock shift OMVs was approximately 4-fold higher than expected, whereas the amounts of the late Group 4 species, G:A and H:A, were roughly 3-fold lower than anticipated (see Fig. S8B in the supplemental material). An identical trend was observed in the OMVs from the 5.8L-to-7.6H shift experiment (Fig. 8B; see Fig. S8B in the supplemental material).

These results indicate that the stochastic model of lipid A incorporation into OMVs seems to hold only for neutral pH/high Mg^2+^ conditions, an environment very favorable for cell growth and not generally associated with other stressors *in vivo*. Changing environmental conditions seem to result in delayed incorporation of newly synthesized species into OMVs. Additionally, exposure to the mildly acidic pH seems to disfavor the incorporation of the highly modified Group 4 lipid A species into OMVs, regardless of whether the cultures experienced long-term exposure to a mildly acidic pH or a recent shift from a mildly acidic pH. These deviations from the stochastic model indicate that other factors influence the regulation of OMV lipid A composition.

## DISCUSSION

The ability of Gram-negative bacteria to rapidly alter the outermost membrane in response to changing environmental conditions is vital to their survival. In this study, we established a protocol for producing reproducible, measurable OM change in wild-type bacteria undergoing physiologically relevant changes in environmental conditions. We performed a quantitative comparison of the structural heterogeneity of the lipid A portion of LPS in the OM and OMV content produced over time.

Considering the relatively low levels of LPS diffusion (7, 24) present in the OM, coupled with the stochastic, patchwork nature of OM biogenesis (11), the Gram-negative OM could theoretically suffer from widespread and/or localized areas of vulnerability during periods of rapid environmental change if the only means of altering LPS structural variants were through the gradual dilution concomitant with normal growth and cell division. Hostile environmental conditions that restrict or lower the cell growth rate could be very detrimental to bacteria in transition to a new ideal membrane composition.

In the neutral pH, high magnesium medium, extensive cross-linking between lipid A phosphate and pyrophosphate residues creates a strong permeability barrier (16). The relatively low production and small average size of OMVs observed under the 7.6H-to-7.6H condition may reflect the robustness of the membrane structure. As the OMV lipid A composition directly mirrored the OM, vesiculation under this condition may be representative of the action of steady-state maintenance processes.

The 7.6H-to-5.8L environmental shift destabilizes the highly cross-linked membrane structure via the loss of divalent cations. Therefore, the progression of the OM lipid A change occurred rapidly in the environmental 7.6H-to-5.8L shift in conditions, with covalently modified species detected at the OM after only 15 min and the percentage of modified species reaching approximately 40% of the total composition after 90 min. Conversely, the modified species decreased from 80% to 50% of the total composition throughout the 90 min of the 5.8L-to-7.6H reverse shift in conditions.

OMVs produced under both changing (7.6H to 5.8L, 5.8L to 7.6H) and steady-state (5.8L to 5.8L) acidic, magnesium-limiting conditions were found to contain lesser amounts of the more highly covalently modified lipid A structures than would be predicted by modeling stochastic production based upon the OM lipid A composition over time. Likewise, the hepta-acylated 1-diphosphate, 4′-phosphate lipid A structure was found to be enriched in the OMVs secreted during the 5.8L-to-5.8L shift, while the hepta-acylated 1,4′-bisphosphate species was enriched in the OMV lipid A content during the 7.6H-to-5.8L shift. The appearance of these trends across disparate environmental shifts (7.6H to 5.8L, 5.8L to 5.8L, and 5.8L to 7.6H) points to the importance of the intrinsic physical properties of different lipid A molecules in the vesiculation process (Fig. 8C; see Table S1 in the supplemental material). Therefore, we considered whether the well-established model of polymorphic regulation of lipid composition could explain some facets of the observed nonstochastic incorporation of lipid A molecules into OMVs under 5.8L conditions.

In the model of polymorphic regulation of membrane lipid composition, the balance between bilayer-promoting and non-bilayer-promoting lipids is closely regulated, maintained throughout disparate environments, and essential for cell viability (25, 26). Nonlamellar types of lipids, which have an overall conical shape and a preference for the hexagonal phase (H_II_), are thought to aid in the formation of nonbilayer structures. Accordingly, both the geometry and associated propensity of individual membrane components to form nonbilayer lipid phases in the membrane are important to vesiculation processes (27).

Considering that 70 to 80% of the inner leaflet phospholipids of the OM are composed of derivatives of PE, a nonbilayer lipid, the intrinsic curvature preference of this leaflet is highly negative (28). This property is likely mirrored in the outer leaflet, where the lipid A molecules must exert an equally powerful negative spontaneous curvature to maintain proper bilayer structure (29). The opposing spontaneous curvature of the OM leaflets results in substantial stored membrane stress, the driving force of transformation to nonbilayer lipid structures (29). Prior to vesicle formation, differences between the elastic properties of the inner and outer leaflets (because of transmembrane asymmetry) initiate curvature generation (29).

As the estimated bulk (68%) of the van der Waals volume of the base 1,4′-bisphosphate hexa-acylated lipid A lies in the hydrocarbon chains (Fig. 8C), we have categorized it as a slightly nonlamellar lipid. Increased preference for the H_II_ phase may occur when the effective head group size decreases, the splay of hydrocarbon chains increases, or the hydrocarbon chain length increases (25). Accordingly, palmitoylation by PagP may serve to increase the nonbilayer propensity of lipid A molecules by increasing both acyl chain length and hydrocarbon splay. The addition of covalent head group modifications could induce the opposite effect through increasing the effective head group size.

Given that the estimated rate of diffusion of an LPS molecule (3 × 10^−6^ nm^2^/s [24]) is much lower than that of phospholipids, the polymorphic model of regulation of lipid composition must be modified to fully explain the behavior of the much larger LPS molecules. If we assume nonideal mixing of the lipid A molecules because of interactions between individual molecules, then the importance of the covalent modifications becomes 2-fold: geometric and electrostatic. It has been previously proposed that the ability of l-Ara4N and pEtN groups to mask anionic phosphate residues may be important in maintaining the stability and bulk electrostatic properties of the membrane in the absence of divalent cations (16). Supporting this assertion, the hexa- and hepta-acylated 1-diphosphate, 4′-phosphate lipid A species possess greater negative charge to volume ratios than other species present under the 5.8L conditions and both structures are also the most prone to secretion via OMVs (Fig. 8C). Taken together, both physical and geometric considerations serve to partially explain the increased release of palmitoylated lipid A species via OMVs and the decreased release of more highly covalently modified lipid A species (Fig. 8C).

The principles of a polymorphic regulation model combined with considerations for nonideal mixing of lipid components can also explain the results of a recent study that observed an accumulation of deacylated lipid A species in the OMVs of LB-grown *S*. Typhimurium overexpressing *pagL* (22). The 3-O-deacylase activity of PagL reduces the area of the hydrophobic cross-section of lipid A, giving it a cylindrical molecular shape (30). Although this shape should be lamella promoting, in the low concentration of divalent cations present in LB medium, this modification may primarily serve to increase the negative charge to total volume ratio of deacylated species, making them more prone to secretion via OMVs in order to maintain the proper electrostatic properties of the OM surface.

As vesicle production is a complex process involving numerous cell envelope components, additional mechanistic factors may be involved in establishing the lipid A content of released OMVs. Notably, the OMP composition of many bacteria has been demonstrated to change in response to pH. In *Salmonella enteritidis*, the abundance of the major OMPs OmpC, OmpF, and OmpA varied dependent upon the medium pH; OmpF and OmpA were both more abundant than OmpC at pH 7, whereas at pH 5 to 6, a new OMP appeared at 26 kDa while OmpF experienced less expression than OmpC and OmpA (31). We postulated that LPS association with OMPs, as observed for OmpF (32), could possibly influence the rate at which certain species are retained in the OM if the OMPs themselves experience various rates of incorporation into OMVs. Differences in the distribution of charged residues present on lipid A head group-proximal regions of OMPs may partially account for the observed retention of more highly modified (and thus less negatively charged) lipid A structures. Despite the small differences in OM-localized protein content that develops during the environmental shifts (see Fig. S3I in the supplemental material), the observed protein composition of OMVs derived from the 90-min samples of either the 7.6H-to-7.6H and 7.6H-to-5.8L shifts or the 5.8L-to-5.8L and 5.8L-to-7.6H shifts did not significantly differ (see Fig. S7B in the supplemental material). Therefore, differential OMP incorporation into OMVs was not likely a major source of the lipid A selectivity or production level differences we observed. However, it is entirely possible that differences in protein content not visible by the methods used in this work may influence the lipid A content retained in the OM and/or released via OMVs.

We next considered that the acidic pH could affect regulatory processes localized in the periplasmic space, resulting in an overall increase in OMV production levels. In previous mechanistic studies, the activity of enzymes that modulate the structure of the peptidoglycan (PG) layer and the activity of proteins that modulate the levels of covalent cross-linking of the PG to the OM via the lipoprotein Lpp have been shown to influence OMV production (33). We found that the total cellular amount of Lpp-PG cross-linking observed under the 7.6H-to-5.8L shift and 7.6H-to-7.6H and 5.8L-to-5.8L mock shift conditions did not differ significantly (see Fig. S7C in the supplemental material). However, this experiment gave no information on whether the Lpp-PG cross-links may be altered in their lateral distribution, allowing for the targeted retention of certain OM areas over others.

The OMV lipid A composition of the 5.8L-to-5.8L condition may have given us further insight into the nature of OM biogenesis. While 50- to 100-nm bursts of protein material being added to the OM have been observed by electron and fluorescence microscopy, the LPS content and protein heterogeneity of these additions remain entirely unknown (11, 34). Because of the low diffusion rate of LPS molecules (3 × 10^−6^ nm^2^/s [24]), our data suggest that certain groups of similarly modified lipid A molecules may be incorporated into the OM in a single burst event.

The relative dearth of covalently modified LPS species in OMVs produced during the 5.8L-to-7.6H shift, the 5.8L-to-5.8L mock shift, and (to a lesser extent) the 7.6H-to-5.8L shift in conditions may reflect the ability of bacteria to maintain a remodeled OM composition despite an environmentally induced increase in OMV production. A multitude of organismal benefits could result from this retention of modified lipid A species in the OM. The resultant masking of the negatively charged phosphate moieties may allow for a more robust permeability barrier in the lower concentration of divalent cations. Unmodified lipid A species, which take less energy to synthesize, may be more energetically favorable for the cell to lose to the OMV functions of OMP maintenance and membrane damage alleviation. Furthermore, the release of less modified and more highly acylated lipid A structures could act to manipulate the host immune system response, whereby the structure most encountered by immune cells would not be representative of the invading cell’s actual membrane composition.

A model based upon the polymorphic regulation of different lipid A structures, combined with the stochastic, patchwork nature of OM biogenesis, may account for the unexpected retention of more highly modified lipid A species in the OM under the mildly acidic environmental conditions. Sensing of these local differences through association with OMPs, periplasmic regulatory protein activity, and/or differential Lpp tethering of the OM to the underlying PG could account for the deviation of observed OMV lipid A content from the stochastic OMV formation model.

## MATERIALS AND METHODS

### Bacterial strains and growth conditions.

The strain used in this work was *Salmonella* Typhimurium 14028S (virulent wild type). Bacteria were grown at 37°C in N-minimal medium [5 mM KCl, 7.5 mM (NH_4_)_2_SO_4_, 0.5 mM K_2_SO_4_, 1 mM KH_2_PO_4_, 0.1 M Tris-HCl, 0.1% Casamino Acids] (35) with 0.4% glucose (14), adjusted to pH 5.8 or 7.6 with HCl, with or without 10 mM MgSO_4_, as indicated (36).

### Shift protocol.

N-minimal medium (either pH 7.6 10 mM MgSO_4_ or pH 5.8 10 µM MgSO_4_, as indicated) was inoculated (1:100 dilution) with bacterial cultures grown overnight (37°C, shaking 200 rpm). Cultures were grown to an OD_600_ of 0.6 and pelleted with a Beckman Avanti J-25 centrifuge (JLA-10.500 rotor; 3,000 × *g*, 7 min, 25°C), and the resulting supernatant was decanted. Cells were thoroughly resuspended in fresh N-minimal medium (either pH 7.6 10 mM MgSO_4_ or pH 5.8 10 µM MgSO_4_, as indicated) and grown at 37°C for 90 min.

### OMV purification.

OMVs were isolated from broth cultures as follows. One-liter cultures were pelleted with a Beckman Avanti J-25 centrifuge (JLA-10.500 rotor, 10,000 × *g*, 10 min, 4°C), and the resulting supernatant was filtered through a 0.45-µm cellulose membrane. The filtered supernatant was then subjected to centrifugation (JLA-16.250 rotor, 38,400 × *g*, 3 h, 4°C) before pellets were resuspended in either Dulbecco’s phosphate-buffered saline supplemented with salt (for SDS-PAGE or other analysis) or 50 mM HEPES, pH 7.4 (for lipid A purification), and frozen at −20°C until utilization. For a large-scale OMV preparation, the cell-free supernatant was first concentrated with a tangential-flow apparatus to reduce the supernatant volume to 750 ml before centrifugation (JLA-16.250 rotor, 38,400 × *g*, 3 h, 4°C).

### Lipid A isolation.

For OMV and WC preparations, salt removal was performed by washing them three times with 50 mM HEPES, pH 7.4. We performed a variant of the protocol of Zhou et al. (15). The samples (1 ml) were lysed with 2:1 methanol-dichloromethane (3 ml) for 1 h at room temperature. Centrifugation (2,000 rpm, 30 min, 25°C) of the samples was followed by washing with 2:1 methanol-dichloromethane and another centrifugation step. The washed, dried (under N_2_) pellet was boiled for 30 min in 12.5 mM sodium acetate (adjusted to pH 4.5 with acetic acid). Lipid A was then isolated via two-phase Bligh/Dyer separation and subsequent washes with fresh neutral upper phase before final drying under N_2_.

### ESI-MS of lipid A samples.

Mass spectra were acquired on a QSTAR-XL quadrupole time-of-flight tandem mass spectrometer (ABI/MDS-Sciex, Foster City, CA) equipped with an ESI source. The spectra were acquired in the negative-ion mode and were largely the accumulation of 60 scans over a range of 500 to 2,500 atomic mass units (37). For MS analysis, the extracted lipids were dissolved in 50 µl of dichloromethane-methanol (2:1, vol/vol) and infused into the ion source at 5 to 10 µl/min. Negative-ion ESI was performed at −4,200 V. Data acquisition and analysis were carried out with AnalystQS software and displayed with mMass tools (38).

### TLC.

Lipid A samples were spotted onto glass-backed silica gel 60 TLC plates and allowed to dry before development in a solvent system consisting of 25:15:4:4 chloroform-methanol-acetic acid-water. Plates were subsequently sprayed with 10% H_2_SO_4_ in ethanol for detection of all species after careful, even heating at 300°C.

### TLC densitometry.

The lanes of each sample run on a TLC plate were set to a common width (0.29) across all plates. After selection of the lanes, the intensity of the bands was plotted by the Gel Analyzer function, with peaks inverted. The area of each peak was defined by a straight line at the base, stretching from the lowest point of the surrounding troughs, before measurement with the Wand (tracing) tool (see Fig. S4 in the supplemental material).

### TEM.

Bacterial cells or OMVs were washed and resuspended in 50 mM HEPES, pH 7.5. Cells were fixed with 4% formaldehyde. Samples were placed on 300-mesh copper grids (Electron Microscopy Sciences) before staining with 1% uranyl acetate (39).

### OMV diameter quantitation.

The OMVs observed on the micrographs were measured from the outermost points of the membrane with the measurement function in the NIH ImageJ software.

### OMV production quantitation.

To quantitate OMV yield, OMV preparations were boiled for 10 min in Laemmli buffer, separated by 15% SDS-PAGE, and stained overnight with SYPRO Ruby Red (Molecular Probes). The gel was fixed for 1 h in a solution of 10% methanol and 7% acetic acid before and after staining. Ruby-stained proteins were visualized under UV light. OmpF/OmpC and OmpA were quantified via densitometry (NIH ImageJ software) (40). A second portion of the OMV preparations was incubated with FM4-64 (Molecular Probes) (3.3 µg/ml in phosphate-buffered saline for 10 min at 37°C). OMVs alone and the FM4-64 probe alone were negative controls. After excitation at 506 nm, emission at 750 nm was measured with a Molecular Devices SpectraMax GeminiXS fluorometer (23). OMV production was normalized by dividing by the number of CFU of each culture per milliliter, and these values were further divided by the OMV production of the control condition to give rise to relative fold OMV production.

## SUPPLEMENTAL MATERIAL

Table S1  Comparison of cellular (e.g., OM) and OMV lipid A compositions after 90-min 7.6H-to-5.8L and 5.8L-to-5.8L shifts in conditions. The *M*_r_ is the calculated molecular weight of the proposed structure; italicized values indicate less certainty in assignments. Table S1, DOCX file, 0.03 MB

Text S1  Supplemental methods used in this study. Download Text S1, DOCX file, 0.03 MB

Figure S1  Previous exposure to mildly acidic low-magnesium conditions increases survival during a PMB challenge. PMB at 0, 4, or 8 µg/ml was administered to cultures that underwent 0 (A), 45 (B), or 90 (C) min in the 7.6H-to-5.8L environmental shift protocol. These cultures were allowed to grow for 0, 2, or 4 h before CFU were counted in order to obtain the percent survival of the treated cultures. Representative results are shown. Download Figure S1, TIF file, 0.6 MB

Figure S2  Environmental shift conditions slightly affect cell growth but not membrane integrity. (A) Growth curves of OD_600_ cell density and CFU measurements from 90 min under the 7.6H-to-7.6H and 7.6H-to-5.8L conditions shown on a log scale. (B) Growth curves of OD_600_ cell density and CFU measurements from 90 min under the 5.8L-to-5.8L and 5.8L-to-7.6H conditions shown on a log scale. (C) Relative fluorescence of cells treated with Sytox Green at different time points of environmental shift protocols compared to that of heat-killed cells under the same conditions. Download Figure S2, TIF file, 1.5 MB

Figure S3  Environmental shift conditions do not affect cell morphology. (A) TEM micrograph of cells grown until directly prior to the environmental shift (*t* = 0). (B) TEM micrograph of cells having undergone 90 min of 7.6H-to-7.6H treatment. (C) TEM micrograph of cells having undergone 90 min of 7.6H-to-5.8L treatment. (D) ImageJ quantification of the lengths and diameters of at least 25 cells chosen at random from three representative micrographs under each condition. (E to H) 2D TLC of GPLs isolated from the OM of cells having undergone 90 min of 7.6H-to-7.6H (E), 7.6H-to-5.8L (F), 5.8L-to-5.8L (G), or 5.8L-to-7.6H (H) treatment. (I) Ruby-stained SDS-PAGE analysis of the OM of cells after 90 min of environmental shift. Lanes: 1, unstained protein ladder (Bio-Rad); 2, 7.6H-to-7.6H OM; 3, 7.6H-to-5.8L OM; 4, 5.8L-to-5.8L OM; 5, 5.8L-to-7.6H OM. Download Figure S3, TIF file, 1.7 MB

Figure S4  Lipid A structures present during the 7.6H-to-5.8L environmental shift with group numbers and TLC densitometry labeling. (A) TLC separation of WC lipid A from cells that have undergone the 7.6H-to-5.8L environmental shift. The bands are individually labeled on the right, and their predicted group numbers are on the far right. (B) Three WC lipid A samples are shown charred and stained with ninhydrin to identify which bands represent nitrogen-modified species (pEtN or L-4-AraN). Lanes: 1, 7.6H-to-5.8L for 75 min; 2, 7.6H-to-7.6H for 90 min; 3, 5.8L-to-5.8L for 90 min. (C) Structures of lipid A species shown with the group numbers assigned to them by H. S. Gibbons et al. (14). Download Figure S4, TIF file, 1.2 MB

Figure S5  TLC densitometry band delineation and charring reproducibility. TLC separation of WC lipid A from cells that have undergone 90 min of the 7.6H-to-7.6H (A) or 7.6H-to-5.8L (B) environmental shift. In each dilution, the bands are individually labeled on the left (A to D or A to F). In panel B, two overlapping peaks are represented by the letters B and C for ease and reproducibility of quantitation. In the dotted rectangle, the gel function of ImageJ is demonstrated, with lines drawn from the troughs between the peaks of each letter grouping. Easily distinguishable noise is also excluded with lines. The area delineated from this process was measured via the wand (tracing) tool. Values were then made proportional to band A before conversion to percent composition, as shown on the right. Dark-colored columns show the values presented in Fig. S6, while light-colored columns show the data obtained from experimental replicates run at a later date. Download Figure S5, TIF file, 1.4 MB

Figure S6  Comparison of OM and OMV lipid A compositions under the 7.6H-to-7.6H, 7.6H-to-5.8L, 5.8L-to-5.8L, and 5.8L-to-7.6H conditions. The data from Fig. 2C (A), 3C (B), 4C (C), and 5C (D) are presented as percentages of the total composition with standard errors. The number of replicates averaged for each time point and the number of TLC plates from which these replicates were gathered are shown below each time point. Download Figure S6, TIF file, 1.6 MB

Figure S7  Evaluation of OMV density, OMV protein content, bacterial Lpp-PG cross-linking levels, and OMV production levels for growth under various environmental conditions. (A) OptiPrep density gradient fractionation of OMVs collected from 7.6H-to-7.6H and 7.6H-to-5.8L conditions was performed. Fractions were collected (1 = lowest density, 12 = highest density), run on SDS-PAGE, and stained with SYPRO Ruby Red (Molecular Probes), and OMV production levels were quantified by OMP densitometry. The level of fluorescence was normalized to the total OMP fluorescence of all of the gradient fractions. (B) Ruby-stained SDS-PAGE analysis of OMVs collected after a 90-min environmental shift. Lanes: 1, unstained protein ladder (Bio-Rad); 2, 7.6H-to-7.6H OMVs; 3, 7.6H-to-5.8L OMVs; 4, 5.8L-to-5.8L OMVs; 5, 5.8L-to-7.6H OMVs. (C) Cross-linked Lpp levels in environmentally shifted cells. Cells that underwent 90 min of the 7.6H-to-7.6H, 7.6H-to-5.8L, and 5.8L-to-5.8L environmental shifts were treated with lysozyme, PG was isolated, and the amount of Lpp copurified with the PG was analyzed by quantitative Western blotting with anti-Lpp antibody (Silhavy Lab). The amount of cross-linked Lpp was normalized to cell pellet weights, and relative amounts are shown. (D and E) Overnight OMV production under conditions varying in pH and Mg^2+^ concentration. Cells were grown overnight under four environmental conditions (7.6H, 7.6L, 5.8H, and 5.8L) before the collection of OMVs. OMV production per CFU relative to the first condition was calculated on the basis of protein content determined by OMP densitometry (D) and on the basis of lipid content determined by FM4-64 fluorescence (E). Download Figure S7, TIF file, 1.5 MB

Figure S8  Comparison of experimentally determined OMV compositions by using theoretical stochastic models. Shown are data from Fig. 8A and B in bar graph form with standard errors. Download Figure S8, TIF file, 1.2 MB
