# Supplementary material for: Outer Membrane Vesicle Production Facilitates LPS Remodeling and Outer Membrane Maintenance in Salmonella during Environmental Transitions
Source: mBio. 2016 Oct 18;7(5):e01532-16. doi: 10.1128/mBio.01532-16 (PMC5082901; doi:10.1128/mBio.01532-16)
Supplement: Table S1 — Comparison of cellular (e.g., OM) and OMV lipid A compositions after 90-min 7.6H-to-5.8L and 5.8L-to-5.8L shifts in conditions. The Mr is the calculated molecular weight of the proposed structure; italicized values indicate less certainty in assignments. [file mbo005163021st1.docx]

**Table S1. Comparison of cellular (e.g. OM) and OMV lipid A compositions after 90 min 7.6H-5.8L and 5.8L-5.8L shift in conditions.**

|  |  |  |  |  |  |  |  | **Acyl chain additions** | |  | **Relative intensity** | |
| --- | --- | --- | --- | --- | --- | --- | --- | --- | --- | --- | --- | --- |
|  |  |  | **Groups at 1- and/or 4'-positions** | | | |  |  |  |  |  |  |
| **Growth condition** | **M_r_^a^** | **[M-H]^-^_p_^b^ (m/z)** | **P ^c^** | **di-P^d^** | **P-PEtN^e^** | **P-L-Ara4N^f^** |  | **C_16:0_^g^** | **OH^h^** |  | **OM^i^** | **OMV^j^** |
|  |  |  |  |  |  |  |  |  |  |  |  |  |
|  |  |  |  |  |  |  |  |  |  |  |  |  |
| 7.6H-5.8L | *1798* | 1775 | 2 |  |  |  |  |  |  |  | 100 | 100 |
|  | 1798 | 1791 | 2 |  |  |  |  |  |  |  | 30 | 42 |
|  | 1798 | 1796 | 2 |  |  |  |  |  |  |  | 57 | 62 |
|  | 1813 | 1811 | 2 |  |  |  |  |  | 1 |  | 30 | 37 |
|  | 1841 | 1838 |  |  | 1 |  |  |  |  |  | 38 | 22 |
|  | 1856 | 1854 |  |  | 1 |  |  |  | 1 |  | 16 | ND |
|  | 1921 | 1918 | 1 |  | 1 |  |  |  |  |  | 84 | 37 |
|  | 1929 | 1926 | 1 |  |  | 1 |  |  |  |  | 15 | ND |
|  | 2036 | 2033 | 2 |  |  |  |  | 1 |  |  | 20 | 18 |
|  | 2053 | 2049 |  |  | 1 | 1 |  |  |  |  | 97 | ND |
|  | 2068 | 2065 |  |  | 1 | 1 |  |  | 1 |  | 36 | ND |
|  |  |  |  |  |  |  |  |  |  |  |  |  |
|  |  |  |  |  |  |  |  |  |  |  |  |  |
| 5.8L-5.8L | 1718 | 1716 | 1 |  |  |  |  |  |  |  | 42 | ND |
|  | 1734 | 1732 | 1 |  |  |  |  |  | 1 |  | 100 | ND |
|  | *1798* | 1751 | 2 |  |  |  |  |  |  |  | 47 | ND |
|  | *1798* | 1767 | 2 |  |  |  |  |  |  |  | 38 | ND |
|  | 1798 | 1795 | 2 |  |  |  |  |  |  |  | <15 | ND |
|  | 1850 | 1847 |  |  |  | 1 |  |  |  |  | 26 | ND |
|  | 1865 | 1863 |  |  |  | 1 |  |  | 1 |  | <15 | 20 |
|  | 1878 | 1882 | 1 | 1 |  |  |  |  |  |  | 34 | 38 |
|  | 1894 | 1898 | 1 | 1 |  |  |  |  | 1 |  | 36 | 39 |
|  | 1957 | 1954 |  |  |  |  |  | 1 |  |  | 25 | <15 |
|  | 2088 | 2086 |  |  |  | 1 |  | 1 |  |  | <15 | 23 |
|  | 2103 | 2101 |  |  |  | 1 |  | 1 | 1 |  | 26 | 37 |
|  | 2117 | 2120 | 1 | 1 |  |  |  | 1 |  |  | <15 | 90 |
|  | 2133 | 2136 | 1 | 1 |  |  |  | 1 | 1 |  | 32 | 100 |
|  | 2160 | 2161 | 1 |  | 1 |  |  | 1 |  |  | 40 | 22 |
|  | 2175 | 2180 | 1 |  | 1 |  |  | 1 | 1 |  | 45 | ND |
|  | 2283 | 2287 |  |  | 2 |  |  | 1 |  |  | 63 | ND |
|  | 2298 | 2303 |  |  | 2 |  |  | 1 | 1 |  | 73 | ND |
|  |  |  |  |  |  |  |  |  |  |  |  |  |

^a^The M_r_ is the calculated molecular weight for the proposed structure; italicized values indicate less certainty in assignments

^b^The [M-H]^-^_p_ is the predicted value for singly charged MS ions

^c^P, unsubstituted monophosphate

^d^di-P, unsubstituted diphosphate; At the 1-position

^e^P-PEtN, diphosphoethanolamine; At either the 1- or 4’-position, but likely at the 1-position when L-Ara4N is present

^f^P-L-Ara4N, phospho-L-Ara4N

^g^C_16:0_, palmitate addition mediated by *pagP*

^h^OH, in 2-hydroxymyristate mediated by *lpxO*

^i^Abundance of <15 indicates very low abundance.

^j^Abundance of ND was not detected above the noise.
