# Supplementary material for: Outer Membrane Vesicle Production Facilitates LPS Remodeling and Outer Membrane Maintenance in Salmonella during Environmental Transitions
Source: mBio. 2016 Oct 18;7(5):e01532-16. doi: 10.1128/mBio.01532-16 (PMC5082901; doi:10.1128/mBio.01532-16)
Supplement: Text S1 — Supplemental methods used in this study. Download [file mbo005163021s1.docx]

**Supplementary methods.**

**Sytox green cell integrity assay.** As in Schwechheimer et al (1), Sytox Green (Invitrogen) was used to assess membrane integrity. Cultures (1 L) were grown and 100 μL was placed in a white 96-well plate (in duplicate). To prepare heat-killed cells for a positive control, 5 mL of bacterial culture was pelleted (10,000 × g, 5 min, room temperature), resuspended in 1 mL sterile N-minimal media, and boiled for 3 min, followed by sterile filtering (0.45-μm Ultra-free spin column filters; Millipore); lysates were diluted 10- to 1,000-fold and 100 μL was placed in a 96-well plate (in duplicate). To each sample, 3 µM Sytox Green was added and the mixture was incubated at room temperature in the dark for 10 min. Measurement of fluorescence (excitation: 500 nm, emission: 550 nm) was performed on a Molecular Devices SpectraMAX GeminiXS spectrometer. The average of the duplicate sample measurements was multiplied by the dilution factor and divided by the OD_600_ of the original culture.

**OM isolation.** Pure OM fractions were isolated via the method of *Dalebroux et al* (2).

**Glycerophospholipid isolation and 2D TLC.** Glycerophospholipids were isolated via standard Bligh & Dyer protocol and separated via the method of *Dalebroux et al* (2).

**Ninhydrin staining.** Staining solution was prepared by dissolving 100 mg ninhydrin in 100 mL water-saturated n-butanol and 3 mL acetic acid. Pink spots were visualized after careful, even heating on a 150°C hotplate.

**Opti prep density gradient fractionation of OMVs.** A step gradient made from OptiPrep 60% stock solution, diluted in OptiPrep diluent buffer was layered into a 15 mL tube (3). The series of volumes and Opti concentrations (final % Optiprep, v:v) of the gradient steps, from bottom to top are as follows: 2 mL of sample in 45%, 1 mL of 40%, 1 mL of 35%, 3 mL of 30%, 3 mL of 25%, and filled to the top with 20%. This was centrifuged (Beckman Optima LE-80K, SW 41 Ti rotor) for 18 hr, 41000xg at 5°C. Fractions of equal volumes were collected by pipetting from the top (Fraction 1= lowest density, Fraction 12=highest density)

**Braun’s lipoprotein (Lpp) quantification.** Peptidoglycan was isolated as described before (1, 4). Lysozyme-digested peptidoglycan samples were analyzed by western blotting, probed with anti-Lpp primary antibody (generously provided by the Silhavy laboratory) (5), and the amount of Lpp associated with the purified PG quantitated by densitometry.

**Supplemental References**

1. **Schwechheimer C, Rodriguez DL, Kuehn MJ.** 2015. NlpI-mediated modulation of outer membrane vesicle production through peptidoglycan dynamics in Escherichia coli. Microbiologyopen doi:10.1002/mbo3.244.

2. **Dalebroux ZD, Matamouros S, Whittington D, Bishop RE, Miller SI.** 2014. PhoPQ regulates acidic glycerophospholipid content of the Salmonella Typhimurium outer membrane. Proc Natl Acad Sci U S A **111:**1963-1968.

3. **Chutkan H, Macdonald I, Manning A, Kuehn MJ.** 2013. Quantitative and qualitative preparations of bacterial outer membrane vesicles. Methods Mol Biol **966:**259-272.

4. **Lam H, Oh DC, Cava F, Takacs CN, Clardy J, de Pedro MA, Waldor MK.** 2009. D-amino acids govern stationary phase cell wall remodeling in bacteria. Science **325:**1552-1555.

5. **Cowles CE, Li Y, Semmelhack MF, Cristea IM, Silhavy TJ.** 2011. The free and bound forms of Lpp occupy distinct subcellular locations in Escherichia coli. Mol Microbiol **79:**1168-1181.
