# Supplementary figures and images for: Outer Membrane Vesicle Production Facilitates LPS Remodeling and Outer Membrane Maintenance in Salmonella during Environmental Transitions
Source: mBio. 2016 Oct 18;7(5):e01532-16. doi: 10.1128/mBio.01532-16 (PMC5082901; doi:10.1128/mBio.01532-16)

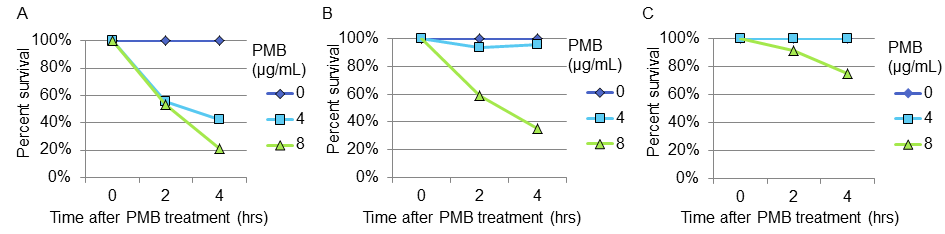

Supplement: Figure S1 — Previous exposure to mildly acidic low-magnesium conditions increases survival during a PMB challenge. PMB at 0, 4, or 8 µg/ml was administered to cultures that underwent 0 (A), 45 (B), or 90 (C) min in the 7.6H-to-5.8L environmental shift protocol. These cultures were allowed to grow for 0, 2, or 4 h before CFU were counted in order to obtain the percent survival of the treated cultures. Representative results are shown. Download [file mbo005163021sf1.tif]

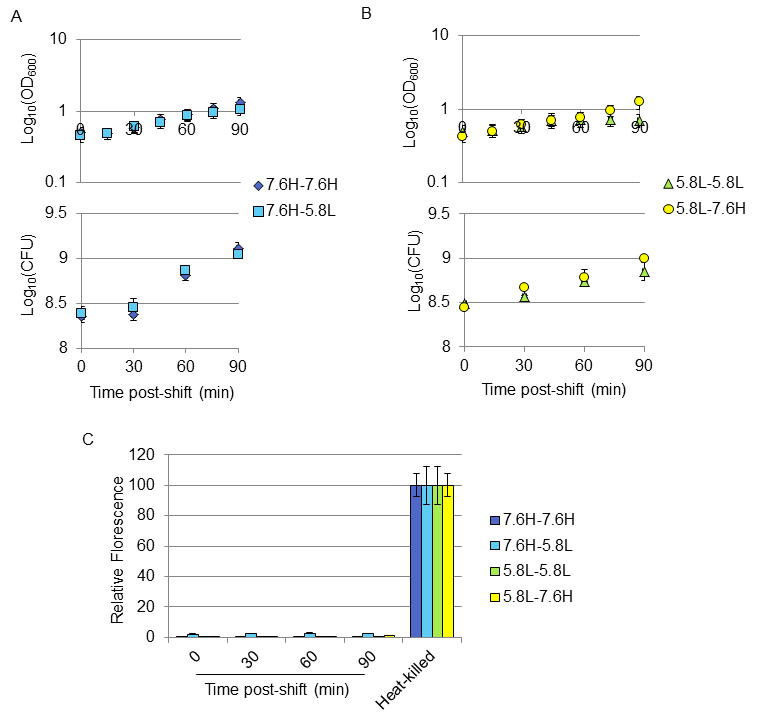

Supplement: Figure S2 — Environmental shift conditions slightly affect cell growth but not membrane integrity. (A) Growth curves of OD600 cell density and CFU measurements from 90 min under the 7.6H-to-7.6H and 7.6H-to-5.8L conditions shown on a log scale. (B) Growth curves of OD600 cell density and CFU measurements from 90 min under the 5.8L-to-5.8L and 5.8L-to-7.6H conditions shown on a log scale. (C) Relative fluorescence of cells treated with Sytox Green at different time points of environmental shift protocols compared to that of heat-killed cells under the same conditions. Download [file mbo005163021sf2.tif]

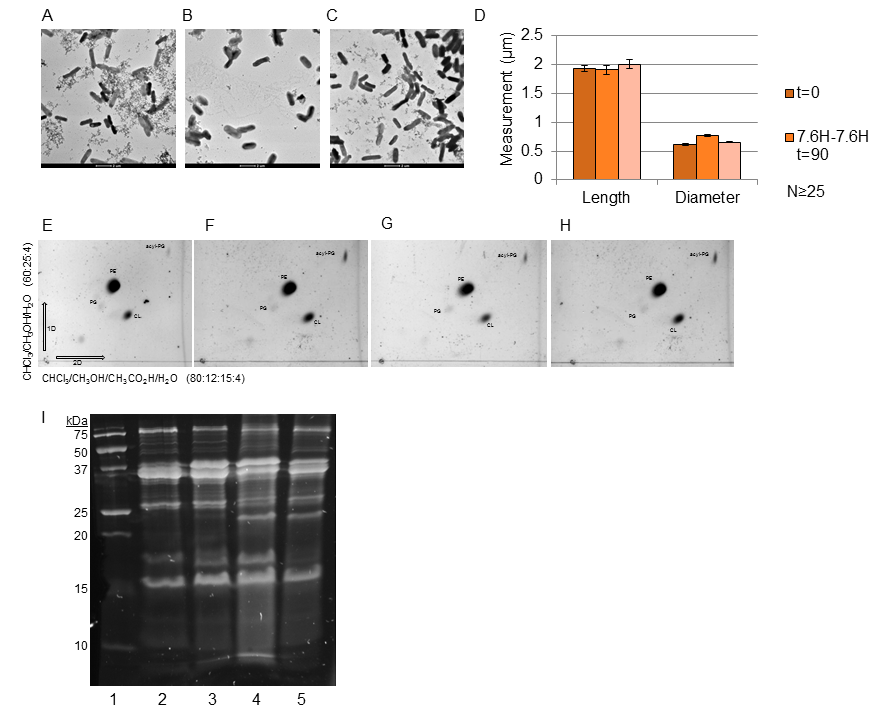

Supplement: Figure S3 — Environmental shift conditions do not affect cell morphology. (A) TEM micrograph of cells grown until directly prior to the environmental shift (t = 0). (B) TEM micrograph of cells having undergone 90 min of 7.6H-to-7.6H treatment. (C) TEM micrograph of cells having undergone 90 min of 7.6H-to-5.8L treatment. (D) ImageJ quantification of the lengths and diameters of at least 25 cells chosen at random from three representative micrographs under each condition. (E to H) 2D TLC of GPLs isolated from the OM of cells having undergone 90 min of 7.6H-to-7.6H (E), 7.6H-to-5.8L (F), 5.8L-to-5.8L (G), or 5.8L-to-7.6H (H) treatment. (I) Ruby-stained SDS-PAGE analysis of the OM of cells after 90 min of environmental shift. Lanes: 1, unstained protein ladder (Bio-Rad); 2, 7.6H-to-7.6H OM; 3, 7.6H-to-5.8L OM; 4, 5.8L-to-5.8L OM; 5, 5.8L-to-7.6H OM. Download [file mbo005163021sf3.tif]

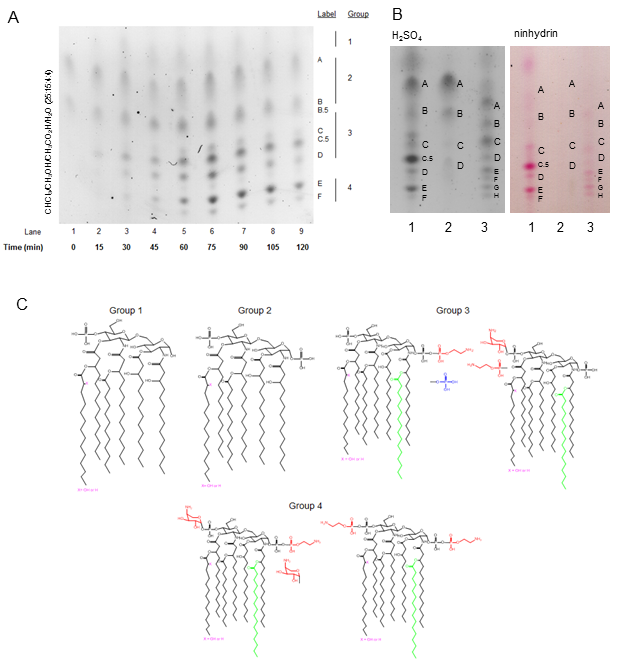

Supplement: Figure S4 — Lipid A structures present during the 7.6H-to-5.8L environmental shift with group numbers and TLC densitometry labeling. (A) TLC separation of WC lipid A from cells that have undergone the 7.6H-to-5.8L environmental shift. The bands are individually labeled on the right, and their predicted group numbers are on the far right. (B) Three WC lipid A samples are shown charred and stained with ninhydrin to identify which bands represent nitrogen-modified species (pEtN or L-4-AraN). Lanes: 1, 7.6H-to-5.8L for 75 min; 2, 7.6H-to-7.6H for 90 min; 3, 5.8L-to-5.8L for 90 min. (C) Structures of lipid A species shown with the group numbers assigned to them by H. S. Gibbons et al. (14). Download [file mbo005163021sf4.tif]

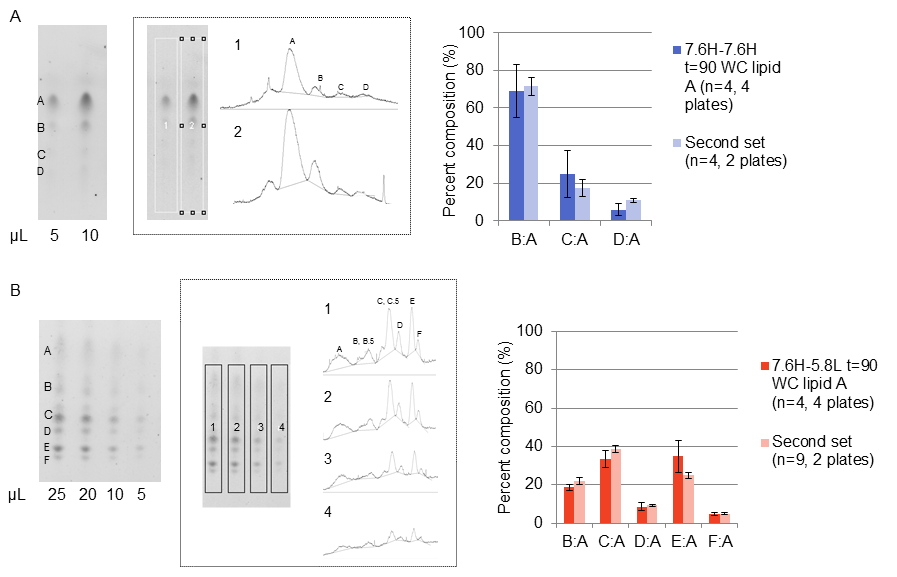

Supplement: Figure S5 — TLC densitometry band delineation and charring reproducibility. TLC separation of WC lipid A from cells that have undergone 90 min of the 7.6H-to-7.6H (A) or 7.6H-to-5.8L (B) environmental shift. In each dilution, the bands are individually labeled on the left (A to D or A to F). In panel B, two overlapping peaks are represented by the letters B and C for ease and reproducibility of quantitation. In the dotted rectangle, the gel function of ImageJ is demonstrated, with lines drawn from the troughs between the peaks of each letter grouping. Easily distinguishable noise is also excluded with lines. The area delineated from this process was measured via the wand (tracing) tool. Values were then made proportional to band A before conversion to percent composition, as shown on the right. Dark-colored columns show the values presented in Fig. S6, while light-colored columns show the data obtained from experimental replicates run at a later date. Download [file mbo005163021sf5.tif]

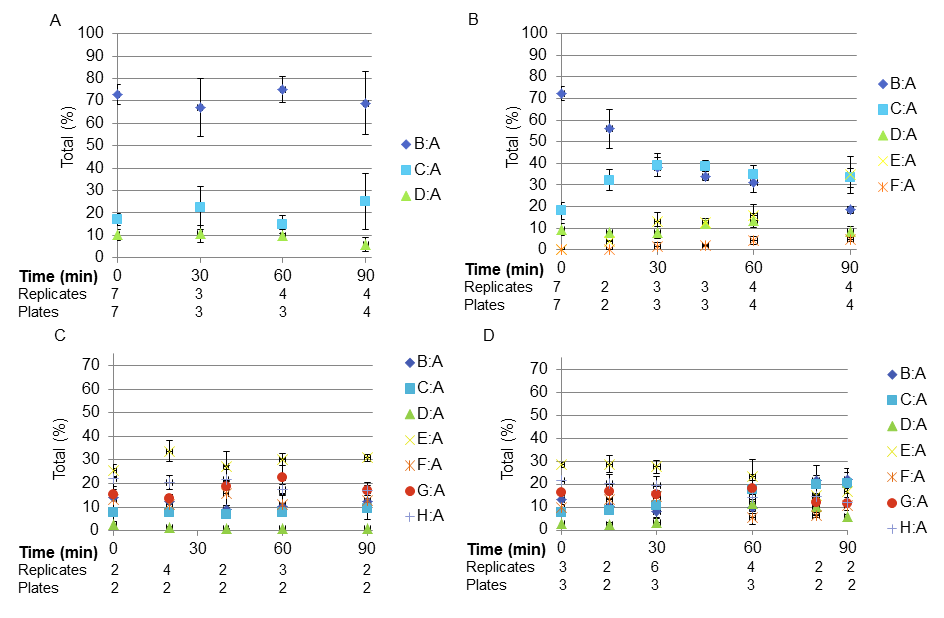

Supplement: Figure S6 — Comparison of OM and OMV lipid A compositions under the 7.6H-to-7.6H, 7.6H-to-5.8L, 5.8L-to-5.8L, and 5.8L-to-7.6H conditions. The data from Fig. 2C (A), 3C (B), 4C (C), and 5C (D) are presented as percentages of the total composition with standard errors. The number of replicates averaged for each time point and the number of TLC plates from which these replicates were gathered are shown below each time point. Download [file mbo005163021sf6.tif]

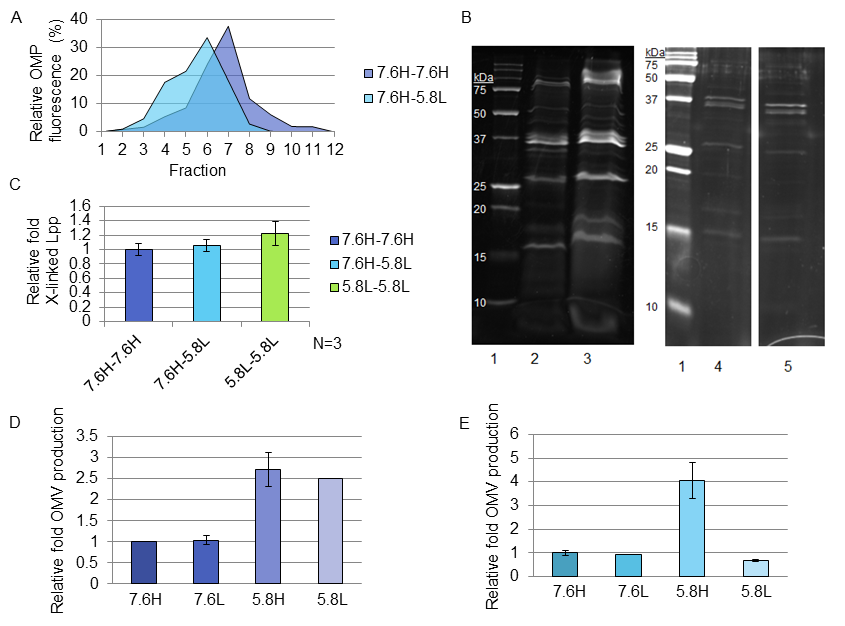

Supplement: Figure S7 — Evaluation of OMV density, OMV protein content, bacterial Lpp-PG cross-linking levels, and OMV production levels for growth under various environmental conditions. (A) OptiPrep density gradient fractionation of OMVs collected from 7.6H-to-7.6H and 7.6H-to-5.8L conditions was performed. Fractions were collected (1 = lowest density, 12 = highest density), run on SDS-PAGE, and stained with SYPRO Ruby Red (Molecular Probes), and OMV production levels were quantified by OMP densitometry. The level of fluorescence was normalized to the total OMP fluorescence of all of the gradient fractions. (B) Ruby-stained SDS-PAGE analysis of OMVs collected after a 90-min environmental shift. Lanes: 1, unstained protein ladder (Bio-Rad); 2, 7.6H-to-7.6H OMVs; 3, 7.6H-to-5.8L OMVs; 4, 5.8L-to-5.8L OMVs; 5, 5.8L-to-7.6H OMVs. (C) Cross-linked Lpp levels in environmentally shifted cells. Cells that underwent 90 min of the 7.6H-to-7.6H, 7.6H-to-5.8L, and 5.8L-to-5.8L environmental shifts were treated with lysozyme, PG was isolated, and the amount of Lpp copurified with the PG was analyzed by quantitative Western blotting with anti-Lpp antibody (Silhavy Lab). The amount of cross-linked Lpp was normalized to cell pellet weights, and relative amounts are shown. (D and E) Overnight OMV production under conditions varying in pH and Mg2+ concentration. Cells were grown overnight under four environmental conditions (7.6H, 7.6L, 5.8H, and 5.8L) before the collection of OMVs. OMV production per CFU relative to the first condition was calculated on the basis of protein content determined by OMP densitometry (D) and on the basis of lipid content determined by FM4-64 fluorescence (E). Download [file mbo005163021sf7.tif]

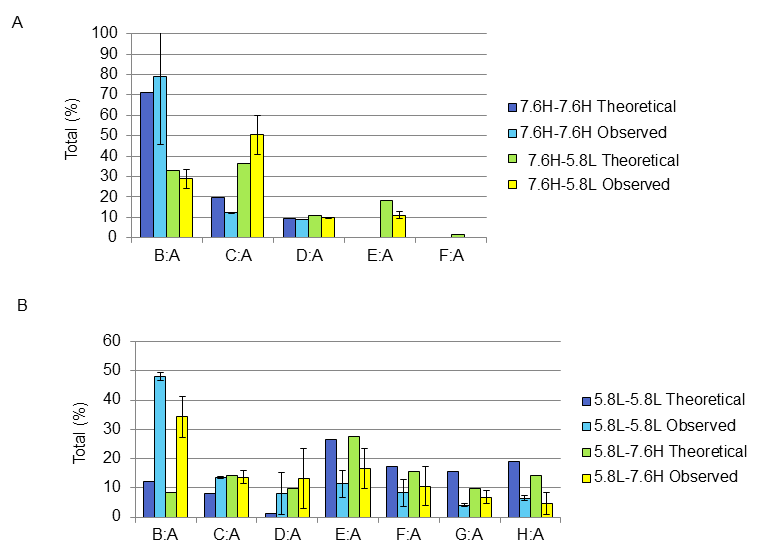

Supplement: Figure S8 — Comparison of experimentally determined OMV compositions by using theoretical stochastic models. Shown are data from Fig. 8A and B in bar graph form with standard errors. Download [file mbo005163021sf8.tif]
